# Efficacy of a digital mental health program for university students: A randomized controlled trial comparing online and hybrid delivery

**DOI:** 10.1016/j.invent.2026.101002

**Published:** 2026-09-26

**Authors:** A. Theurel, M.-A. Martinie, I. Krifa, A. Witt, A. Fournier, Z.-A. Mathieu, M. Faioes Macedo, S. Le Vigouroux, V. Flaudias, I. Millot, L. Bègue, R. Shankland

**Affiliations:** a Univ. Grenoble Alpes, LIP/PC2S, 38000, Grenoble, France; b Centre de Recherches sur la Cognition et l'Apprentissage (CeRCA), Poitiers University, Poitiers, France; c Université Lumière Lyon 2, Laboratoire DIPHE, Lyon, France; d Université Bourgogne Europe, CNRS, LEAD UMR5022, 21000, Dijon, France; e Laboratoire Psy-DREPI, Université Bourgogne Europe, Dijon, France; f Promotion-Santé BFC, Dijon, France; g Laboratoire Activités Physiques et Sportives et processus PSYchologiques: recherches sur les Vulnérabilités -, Nîmes, Université, France; h Laboratoire de Psychologie Sociale et Cognitive (LAPSCO – UMR 6024), Université Clermont Auvergne, Clermont-Ferrand, France; i Institut Universitaire de France, France

**Keywords:** Digital mental health intervention, University students, Psychological distress, Well-being, Emotion regulation, Mental health promotion

## Abstract

University students are a high-risk population for mental health problems. However they often face barriers to care. Digital Mental Health Interventions are scalable tools to support well-being, yet evidence regarding optimal delivery formats remains mixed. This study assessed the efficacy of *ETUCARE*, an 8-week multicomponent digital program comparing online-only and hybrid (online + in-person workshops) delivery formats**.**

A three-arm randomized controlled trial was conducted among 1064 French university students assigned to an online-only (*n* = 460), a hybrid (*n* = 235), or a waitlist control group (*n* = 369). Self-report measures were collected at baseline (T1), post-intervention (T2), and 8-week follow-up (T3). Outcomes included psychological distress, well-being, emotion regulation, insomnia, and alcohol use. Linear mixed-effects models examined time × group effects. Clinical transitions were analyzed using multinomial logistic regressions and chi-square tests.

Significant time × group interactions indicated more favorable change trajectories for psychological distress, emotion regulation, insomnia, and several well-being dimensions in both intervention groups than in the waitlist control group. No consistent differences were observed between delivery formats on global outcomes, and no differential intervention effect was found for alcohol use. Sensitivity analyses showed that program completion contributed additively to some outcomes without accounting for group-related differences. Exploratory clinical transition analyses suggested an increased likelihood of remission for psychological distress and insomnia in both intervention groups, with greater symptom improvement in the hybrid format among students with higher baseline severity, and a possible protective effect of the online-only format against symptom aggravation at post-test, an effect not maintained at follow-up.

ETUCARE showed small benefits over the waitlist condition for psychological distress, insomnia, emotion regulation, and selected dimensions of psychological well-being. The broadly comparable outcomes of the two delivery formats suggest that the online-only format may provide a more scalable option for universal student mental health promotion.

**Trial registration:**

This trial was not prospectively registered.

**Ethics approval:**

The study was approved by the Ethics Committee of Nantes University (IRB No. IORG0011023, Ref. 06042023–2). All participants provided informed consent before enrolment.

## Highlights

• An 8-week digital program improved students' mental health across multiple outcomes
• Online-only and hybrid formats showed comparable overall effectiveness
• Hybrid delivery benefited students with higher baseline symptom severity
• Online-only format linked to less aggravation at post-test
• Completion had additive but limited effects on mental health outcomes

## 1 Introduction

Emerging adulthood (18–29 years) is a critical developmental period characterized by major biological, psychological, and social transitions, including identity formation, increasing autonomy, and key life decisions related to education, relationships, and career paths (Arnett et al., 2014; Lesener et al., 2020). This stage also involves ongoing neurodevelopmental maturation of executive and emotional regulatory systems, alongside significant social reorganization such as residential mobility and the reshaping of social networks, which may increase both opportunities and vulnerability (Lebel and Beaulieu, 2011; Telzer et al., 2023; Worthman and Trang, 2018; Orygen, 2017).

Entering higher education further intensifies these developmental challenges by adding academic, financial, and social stressors, including increased workload, performance pressure, social isolation, and adaptation to new institutional demands (Basson and Rothmann, 2019; Mertens et al., 2020). As a result, university students must navigate multiple concurrent transitions that place high demands on emotional, cognitive, and social resources. Young adulthood, particularly within university settings, is therefore considered a global public health priority due to elevated vulnerability to mental health disorders (Fusar-Poli et al., 2021; Solmi et al., 2022). Even prior to the COVID-19 pandemic, students exhibited high rates of depression, anxiety, suicidal ideation, and substance use (Auerbach et al., 2018; Bruffaerts et al., 2019; Mortier et al., 2018), a burden that has since been exacerbated by broader societal stressors such as economic instability, climate anxiety, and the pandemic itself (Hossain et al., 2022; van Nieuwenhuizen et al., 2021). This combination of risk also makes this period a critical window for preventive mental health interventions tailored to developmental needs.

Despite increased needs, mental health services remain overstretched, with long wait times, underutilization of care, and rising reports of psychological distress among youth (Edbrooke-Childs and Deighton, 2020; Xiong et al., 2020). Despite the rising demand for psychological support in university settings, many students remain underserved due to multiple barriers: limited availability of on-campus counselling, long waitlists, lack of awareness, fear of stigma, and preferences for self-help approaches (Lipson et al., 2021; Radez et al., 2021; Theurel and Witt, 2022).

In this context, digital mental health interventions (DMHIs) have emerged as scalable and accessible tools for mental health promotion and early intervention among young people (Grist et al., 2019; Lattie et al., 2019; Lehtimaki et al., 2021; Linardon et al., 2019). DMHIs encompass diverse formats (e.g., mobile apps, web-based programs, chatbots, virtual coaching) and are commonly grounded in evidence-based approaches such as cognitive behavioural therapy, mindfulness, and positive psychology (Eisenstadt et al., 2021; Bevan Jones et al., 2023). Evidence indicates that DMHIs yield small to moderate effects on psychological symptoms, well-being, and health behaviours, including sleep and substance use (Lehtimaki et al., 2021; Bennett et al., 2020). However, their real-world impact is often limited by low engagement, high attrition, and poor adherence (Bear et al., 2024; Linardon and Fuller-Tyszkiewicz, 2020; Potts et al., 2025). Importantly, higher engagement is consistently associated with greater clinical improvement across conditions and formats, highlighting engagement as a core mechanism of efficacy rather than a simple usage indicator (Gan et al., 2021).

Hybrid interventions combining digital tools with human support have been proposed as a promising strategy to address engagement and adherence challenges. Meta-analyses consistently show that the inclusion of human support (whether minimal or structured) enhances engagement, adherence, and clinical effectiveness (Lipschitz et al., 2023; Mohr et al., 2011; Moshe et al., 2021). Guided interventions tend to outperform self-guided formats, particularly among individuals with higher symptom severity, although effect sizes vary across studies (Grist et al., 2019; Gan et al., 2021; Werntz et al., 2023). Evidence further suggests that structured, proactive, and integrated support is more effective than optional or passive guidance. Qualitative research indicates that hybrid formats may be especially well suited to university students, offering a balance between the flexibility of digital access and the motivational, emotional, and relational benefits of interpersonal interaction (Garrido et al., 2019).

In summary, DMHIs have demonstrated efficacy in improving mental well-being and reducing depression and anxiety in young people. However, evidence remains limited for French university students, and few well-powered three-arm randomized controlled trials have directly compared hybrid and fully digital formats while systematically reporting engagement, completion, and attrition. Multicomponent programs may be particularly relevant in university settings because students frequently experience overlapping difficulties involving psychological distress, sleep, emotion regulation, and health behaviours. By combining psychoeducation with cognitive-behavioural, positive psychology, mindfulness, and lifestyle-based strategies, such programs may address both symptoms and protective psychological resources (Przybylko et al., 2021). Nevertheless, most existing interventions focus on isolated outcomes, and rigorous evaluations of integrated approaches remain limited.

ETUCARE is a multicomponent digital mental health promotion program integrating psychoeducation, self-monitoring, and brief practical activities. A preliminary randomized feasibility study involving 103 university students (intervention: *n* = 53; control: *n* = 50) found higher psychological well-being and lower psychological distress, anxiety, and alcohol consumption at post-test in the intervention group (Theurel et al., 2022). However, this initial study involved a small sample, evaluated only the online format, and did not include longer-term follow-up. The present study therefore evaluated ETUCARE in a large three-arm randomized controlled trial comparing online-only and hybrid delivery with a waitlist control condition across psychological distress, well-being, emotion regulation, insomnia, and alcohol use.

## 2 Method

### 2.1 Trial design

This study was a three-arm randomized controlled trial evaluating the efficacy of the ETUCARE digital mental health intervention for university students. Participants were allocated to one of three conditions: (1) an online-only self-guided intervention, (2) a hybrid intervention combining online modules with weekly in-person group workshops, or (3) a wait-list control group. Self-report assessments were administered at baseline (T1), post-intervention at 8 weeks (T2), and at 2-month follow-up (T3). An a priori power analysis conducted with G*Power 3.1.9.4 (repeated-measures ANOVA, within–between interaction; effect size f = 0.14, α = 0.05, power = 0.90) indicated that approximately 330 participants were required. Given the high attrition typically observed in DMHIs (Potts et al., 2025), a target sample size of at least 825 participants was set to ensure adequate power across time points.

### 2.2 Participants, recruitment and procedure

Eligible participants were university students aged 18 years or older, enrolled in one of these seven French universities (Université Lumière Lyon 2, Université de Bourgogne-Franche-Comté, Université Claude Bernard Lyon 1, Université Grenoble Alpes, Université de Poitiers, Nîmes Université, Université de Nantes), fluent in French, and with internet access. Exclusion criteria included prior participation in the ETUCARE program.

Participants were recruited via university health services, institutional mailing lists, campus posters, and in-class announcements. Recruitment and intervention delivery were organized across four successive waves: February–May 2023, October–December 2023, February–May 2024, and October–December 2024. Study materials described ETUCARE as a universal 8-week program designed to support students' well-being and academic experience, rather than as treatment for a specific mental health or sleep disorder. Recruitment materials did not specifically target students experiencing psychological distress, insomnia, or low well-being, and no minimum symptom-severity threshold was required. Interested students accessed a dedicated website, provided electronic informed consent via SurveyMonkey, and completed the baseline assessment (T1).

Participants were initially randomized in a 1:1:1 ratio to the waitlist control, online-only, or hybrid condition using an automated procedure stratified by academic sector. The hybrid condition had a fixed capacity determined by the number and size of facilitated workshops. Once all hybrid places had been filled, recruitment remained open and subsequent participants were randomly allocated in a 1:1 ratio between the online-only and control conditions. Assignment therefore remained automated and random among the conditions available at enrolment, resulting in unequal final group sizes. Participants allocated to an intervention condition received access to ETUCARE for 8 weeks, with one module released each week and accompanying email reminders.Participants in the hybrid condition additionally attended eight weekly, in-person group workshops (1.5 h each) delivered on campus. No financial incentives were provided. Post-intervention (T2) and two-month follow-up (T3) assessments were administered online. After completion of T3, participants in the wait-list control group were granted access to the ETUCARE program. Within each wave, delivery was organized in predefined cohorts following a common assessment and intervention schedule. T.

### 2.3 Intervention

The ETUCARE program has been described in detail in a previously published article (Theurel et al., 2022). A full description is provided in Supplementary File S1 to support the understanding of the intervention components relevant to this study.

#### 2.3.1 ETUCARE online intervention

The ETUCARE Online program is an 8-week, web-based mental health intervention co-designed with university students. It comprises eight sequential modules (≈45 min each), each addressing a core theme relevant to student mental health (e.g., stress management, emotion regulation, procrastination, sleep, self-esteem). Modules include psychoeducational content delivered through animated presentations, videos, and audio materials, brief self-assessments (e.g., sleep habits, stressors), and short practical exercises (5–15 min) grounded in cognitive-behavioural therapy (CBT) and positive psychology. Modules were completed sequentially, with access to subsequent content contingent on prior completion, although participants could revisit earlier modules at any time. The online format did not include face-to-face interaction. Weekly semi-personalized emails were sent to introduce module content and encourage engagement.

#### 2.3.2 ETUCARE hybrid intervention

The ETUCARE Hybrid intervention combined the online program with eight weekly in-person group workshops (1.5 h each), delivered on campus in groups of 10–15 students. Workshops were designed to reinforce online content through group discussions, experiential activities, and collaborative practice. Delivery was coordinated locally by site-specific academic leads and facilitated by trained mental health professionals or academic staff with clinical expertise. Facilitators completed a standardized 3-day training prior to implementation. Intervention fidelity was supported through a structured trainer's manual and a participant booklet including session summaries and exercises, ensuring consistency across sites while allowing participant engagement and reflective practice.

### 2.4 Measures

All questionnaires were administered in French, using validated French-language versions when available. At baseline, participants also reported sociodemographic and academic information, including age, gender, university site, field of study, level of study and whether they held paid employment alongside their studies.

#### 2.4.1 Depression, anxiety, and stress scale – 21 items (DASS-21)

The DASS-21 (Lovibond and Lovibond, 1995) was used to assess three dimensions of psychological distress: depression, anxiety, and stress. The DASS-21 includes 21 items divided into three subscales. Each subscale consists of seven items rated on a 4-point Likert scale (0 = did not apply to me at all to 3 = applied to me most of the time). An example item is “I felt that I had nothing to look forward to” (depression). The DASS-21 demonstrated excellent internal consistency for the total score (α = 0.91), with good reliability for depression (α = 0.87), anxiety (α = 0.81), and stress (α = 0.82).

#### 2.4.2 Well-being (PERMA Profiler)

Well-being was measured using the PERMA Profiler (Butler and Kern, 2016), a 23-item self-report instrument assessing seven dimensions of well-being derived from Seligman's PERMA model: Positive Emotion, Engagement, Relationships, Meaning, Accomplishment, Negative Emotion, and Health. Each subscale includes 3 items rated on an 11-point Likert scale (0−10). An example item is “In general, to what extent do you lead a purposeful and meaningful life?” (Meaning). Two additional items assess loneliness and overall happiness. In the present study, the PERMA Profiler showed good overall internal consistency (α = 0.84), with subscale reliabilities ranging from acceptable to excellent (α = 0.63–0.91).

#### 2.4.3 Emotion regulation skills questionnaire (ERSQ)

The French version of the ERSQ (Nardelli et al., 2024) is a 27-item self-report measure assessing adaptive emotion regulation skills, based on the Adaptive Coping with Emotions (ACE) model. It encompasses nine subscales (3 items each): emotional awareness, bodily sensations, clarity of emotions, understanding of emotions, modification of emotions, acceptance of emotions, tolerance of distress, emotional confrontation, and self-support. All Items are rated on a 5-point Likert scale (0 = not at all, 4 = almost always). An example item is “I was able to influence my negative emotions” (emotion modification). The ERSQ demonstrated excellent internal consistency for the total score (α = 0.92), with acceptable to good reliability across subscales (α = 0.62–0.82).

#### 2.4.4 Insomnia severity index (ISI)

The ISI (Bastien et al., 2001) is a 7-item self-report instrument assessing the nature, severity, and impact of insomnia. Items cover difficulties with sleep onset, sleep maintenance, early morning awakening, satisfaction with current sleep pattern, interference with daily functioning, noticeability of sleep problems by others, and distress caused by sleep difficulties. Each item is rated on a 5-point Likert scale (0 = no problem, 4 = very severe problem), resulting in a total score ranging from 0 to 28, with higher scores indicating greater insomnia severity. The ISI showed good internal consistency in the present sample (α = 0.84).

#### 2.4.5 Alcohol use disorders identification test (AUDIT)

The AUDIT (Babor et al., 1992) is a 10-item screening tool to identify hazardous and harmful alcohol consumption. The instrument assesses three domains: alcohol consumption (items 1–3), drinking behaviour and dependence (items 4–6), and alcohol-related problems (items 7–10). Total scores range from 0 to 40, with higher scores indicating greater alcohol-related risk. Internal consistency was acceptable in the present sample (α = 0.80), consistent with previous findings in young adult populations (Shevlin and Smith, 2007).

### 2.5 Statistical analyses

Primary analyses followed the intention-to-treat principle, with all 1064 randomized participants retained in the ITT dataset irrespective of intervention initiation or completion. Linear mixed-effects models were used to examine changes in psychological distress (DASS-21), insomnia (ISI), alcohol use (AUDIT), well-being (PERMAH), and emotion regulation skills (ERSQ) across T1, T2, and T3, and to test whether change trajectories differed by intervention format (online-only vs. hybrid). Repeated observations were nested within participants, with a random intercept for participant ID and fixed effects for time, group, and their interaction. Models were estimated using restricted maximum likelihood and incorporated all available outcome observations.

Analyses were conducted in Jamovi (version 2.3.28) using the Mixed Models module. Mixed-effects models allowed participants with incomplete post-intervention or follow-up data to contribute their available observations under the Missing At Random (MAR) assumption. Missingness was examined using logistic regression models assessing associations between assessment completion and observed variables, including gender and assessment time; these associations supported the plausibility of MAR. Attrition reduced the information and precision available at later assessments but did not lead to automatic participant exclusion or replacement of missing scores. Model adequacy was evaluated using AIC, BIC, residual diagnostics, and intraclass correlation coefficients.

Inclusion in the primary analyses was not conditional on program initiation, completion of a minimum number of online modules, or attendance at a minimum number of hybrid workshops. There was therefore no maximum allowable number of missed modules or sessions for participants to remain in the ITT analyses. Adherence indicators were reported separately, and the percentage of completed modules was examined as a continuous covariate in sensitivity analyses.

Clinically meaningful change was assessed using categorical transition analyses for psychological distress, insomnia, and alcohol use. Participants were classified into four trajectories (stable, improvement, remission, aggravation), and multinomial logistic regression models examined transitions as a function of intervention group and baseline severity. Chi-square tests were used to compare transition distributions across groups. Full model specifications and clinical cutoffs are provided in Supplementary File S2.

Sensitivity analyses were conducted by adding the percentage of completed modules as a continuous covariate to the mixed-effects models (Supplementary File S3), in order to assess whether group differences in change trajectories persisted after accounting for engagement intensity. Completion was included as a fixed effect alongside time and group, and changes in model fit (AIC, BIC, marginal and conditional R (Lesener et al., 2020)) and Time × Group interactions were examined.

Partial eta squared values were interpreted using conventional benchmarks of approximately 0.01 for a small effect, 0.06 for a medium effect, and 0.14 for a large effect.

The datasets analyzed during the current study are available in the Open Science Framework (OSF) repository at: https://osf.io/5jhcz/overview?view_only=0f8c753d58864fc3b0b26aa1abc01df4

## 3 Results

### 3.1 Sample characteristics and baseline equivalence

The participant flow and attrition rates are summarized in Fig. 1

**Fig. 1:**
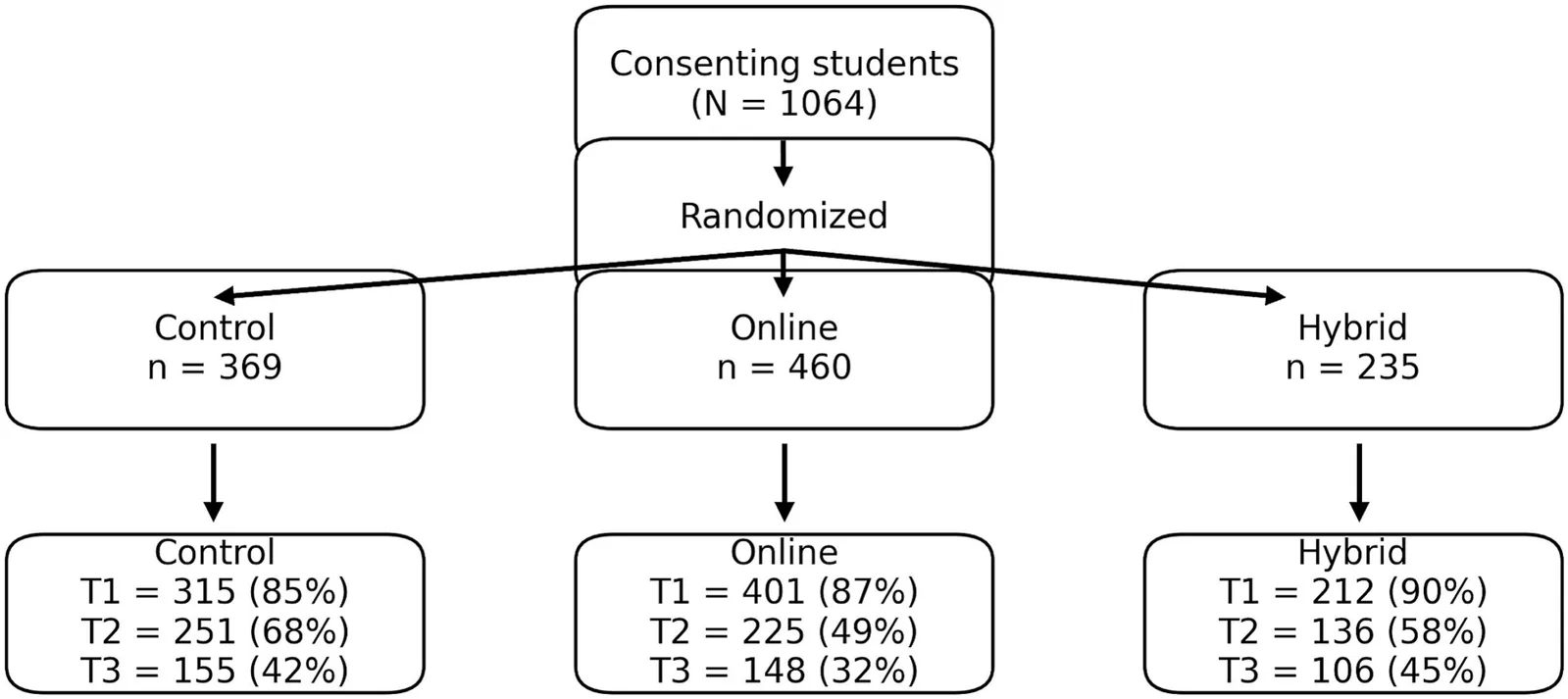
Participant allocation and assessment completion across study conditions.

A total of 1064 consenting students were randomized: 369 to the waitlist control condition, 460 to the online-only condition, and 235 to the hybrid condition. Of these, 928 provided baseline outcome data at T1 (control: *n* = 315; online-only: *n* = 401; hybrid: *n* = 212). Sociodemographic information was available for 911 participants, whose mean age was 21.13 years; 794 were women (87.2%), and 610 were enrolled in psychology-related fields (66.9%). Baseline equivalence between the three study groups (control, online-only, hybrid) was examined using one-way ANOVAs on all primary and secondary outcome measures and support a strong equivalence of psychological profiles across groups at study entry (Table S1).

### 3.2 Participant engagement and adherence

Engagement was assessed through program initiation, completion, and dropout. Initiation rates were high and comparable across groups (78% online vs. 82% hybrid, *z* = 1.19, *p* = .23). Full completion of the eight modules did not differ significantly between conditions (55% online vs. 59% hybrid, *z* = −0.99, *p* = .32), nor among participants who accessed the platform (70% vs. 72%, *z* = −0.37, *p* = .71). In the hybrid group, workshop attendance was high, with 86% attending at least one session and 60% completing all eight; overall, 52% completed the full hybrid pathway. Dropout rates were similar across groups (22% online vs. 18% hybrid, *z* = 1.19, *p* = .23). Overall, both formats showed good engagement and adherence, supporting the feasibility of digital and blended interventions in university settings.

### 3.3 Impact of ETUCARE intervention on psychological outcomes: Main effects

Analyses were conducted on a total sample of 1064 students (control: *n* = 369; online-only: *n* = 460; hybrid: *n* = 235). Descriptive statistics are presented in Table 1. Model fit indices for all linear mixed-effects models are presented in Table 2. Overall, models showed satisfactory adjustment across outcomes. Conditional R^2^ values were consistently high, ranging from 0.42 to 0.86, indicating that a substantial proportion of variance was explained when accounting for both fixed effects and between-participant variability. In contrast, marginal R^2^ values were modest (0.01–0.06), reflecting the relatively small but meaningful contribution of fixed effects in the context of marked inter-individual heterogeneity. Intraclass correlation coefficients (ICCs) were moderate to high across outcomes (0.40–0.86), confirming strong within-person dependency over time and supporting the inclusion of random intercepts. ICCs were particularly elevated for alcohol use and well-being-related outcomes, suggesting that baseline individual differences accounted for a large share of variance in these domains. Across all outcomes, AIC and BIC values indicated adequate model parsimony, with interaction models providing improved fit relative to main-effects-only specifications. Taken together, these indices support the robustness of the modeling approach and the interpretability of the intervention effects reported below. Table 3 presents the omnibus tests from the linear mixed-effects models for all primary and secondary outcomes, reporting the main effects of time, group, and their interaction (time × group), along with associated degrees of freedom, F values, *p*-values, and partial eta squared (η^2^p) effect sizesDetailed post hoc comparisons and estimated marginal mean differences are presented in Supplementary Tables S2–S7**.**

#### 3.3.1 Effects of the intervention on mental health outcomes

##### 3.3.1.1 Psychological distress (DASS-21 total and subscales)

Linear mixed-effects models (Table 3) revealed significant main effects of time for overall psychological distress and all DASS-21 subscales (depression, anxiety, and stress), indicating a general reduction in symptoms across the study period (all *ps* < 0.001). Significant group effects were also observed, reflecting lower overall symptom levels in both intervention groups compared to the control group. Importantly, robust time × group interactions were found for psychological distress and each subscale (all *ps* < 0.001), indicating differential symptom trajectories across conditions.

**Table 1:** Mean (and Standard Errors) of our variable of interest by Group and Time.

	Control			Hybrid			Online		
	T1	T2	T3	T1	T2	T3	T1	T2	T3
Psychological distress	23.40 (0.63)	22.20 (0.67)	20.80 (0.77)	22.20 (0.77)	17.70 (0.88)	16.50 (0.92)	23.60 (0.56)	17.00 (0.66)	16.40 (0.73)
Depression	8.24 (0.26)	7.45 (0.28)	7.11 (0.33)	7.70 (0.32)	6.05 (0.37)	5.81 (0.39)	8.24 (0.23)	5.84 (0.28)	5.74 (0.31)
Anxiety	6.45 (0.23)	6.21 (0.25)	5.80 (0.28)	6.11 (0.28)	4.97 (0.32)	4.59 (0.34)	6.46 (0.21)	4.61 (0.24)	4.50 (0.27)
Stress	8.72 (0.23)	8.59 (0.25)	7.87 (0.29)	8.39 (0.28)	6.73 (0.33)	6.10 (0.35)	8.93 (0.20)	6.56 (0.25)	6.20 (0.28)
Well-being	107 (1.61)	109 (1.68)	114 (1.86)	109 (1.97)	113 (2.17)	116 (2.26)	106 (1.41)	115 (1.61)	116 (1.75)
Positive emotions	17.2 (0.33)	17.6 (0.35)	18.5 (0.39)	17.2 (0.41)	18.2 (0.45)	18.6 (0.47)	16.8 (0.29)	18.7 (0.34)	19.0 (0.37)
Engagement	18.3 (0.31)	18.2 (0.33)	19.4 (0.38)	18.9 (0.38)	18.8 (0.44)	19.5 (0.46)	18.5 (0.28)	19.4 (0.33)	20.1 (0.37)
Relationships	18.9 (0.36)	19.4 (0.38)	19.9 (0.43)	19.4 (0.44)	20.1 (0.50)	20.1 (0.52)	18.8 (0.32)	20.0 (0.37)	19.9 (0.41)
Meaning	17.6 (0.40)	18.2 (0.42)	18.9 (0.47)	18.4 (0.49)	19.5 (0.54)	19.7 (0.57)	17.4 (0.35)	19.4 (0.40)	19.0 (0.44)
Accomplishment	17.1 (0.31)	17.6 (0.33)	18.7 (0.37)	17.1 (0.38)	18.2 (0.42)	19.1 (0.45)	16.8 (0.27)	18.6 (0.32)	19.1 (0.35)
Health	17.8 (0.36)	18.1 (0.38)	18.2 (0.43)	17.8 (0.44)	17.9 (0.49)	18.9 (0.52)	17.5 (0.32)	18.5 (0.37)	19.3 (0.40)
Loneliness	4.98 (0.17)	4.89 (0.18)	4.84 (0.21)	5.10 (0.21)	4.57 (0.24)	5.18 (0.26)	5.04 (0.15)	4.63 (0.18)	4.20 (0.21)
Negative emotions	16.0 (0.31)	15.6 (0.33)	15.2 (0.37)	15.5 (0.38)	14.0 (0.43)	13.6 (0.45)	16.0 (0.27)	13.5 (0.32)	13.5 (0.35)
Emotion regulation	61.50 (0.94)	64 (1)	68.80 (1.16)	61 (1.15)	69.4 (1.32)	69.6 (1.40)	60 (0.83)	68.90 (0.99)	70.90 (1.11)
Emotional awareness	7.17 (0.13)	7.33 (0.15)	7.65 (0.65)	6.82 (0.16)	7.93 (0.19)	7.93 (0.21)	6.84 (0.12)	7.90 (0.15)	8.09 (0.17)
Modification	5.92 (0.14)	6.16 (0.15)	6.55 (0.18)	6.19 (0.17)	6.67 (0.20)	6.67 (0.21)	5.95 (0.12)	7.00 (0.15)	7.14 (0.14)
Understanding	7.45 (0.15)	7.73 (0.16)	8.36 (0.19)	7.10 (0.18)	8.52 (0.21)	8.08 (0.23)	7.15 (0.13)	8.14 (0.16)	8.34 (0.18)
Tolerance	6.03 (0.15)	6.32 (0.17)	6.95 (0.17)	6.13 (0.13)	7.25 (0.22)	7.09 (0.24)	5.85 (0.14)	6.96 (0.17)	7.34 (0.19)
Acceptance	6.38 (0.14)	6.63 (0.15)	7.00 (0.18)	6.44 (0.17)	7.39 (0.20)	7.31 (0.21)	6.16 (0.12)	7.20 (0.15)	7.36 (0.17)
Clarity	7.09 (0.15)	7.48 (0.16)	8.12 (0.19)	6.87 (0.19)	7.95 (0.22)	8.02 (0.23)	6.93 (0.14)	8.02 (0.16)	8.12 (0.18)
Sensation	7.18 (0.16)	7.49 (0.17)	7.81 (0.20)	6.66 (0.19)	7.62 (0.23)	7.80 (0.24)	6.74 (0.14)	7.70 (0.17)	7.90 (0.90)
Confrontation	6.87 (0.15)	7.15 (0.16)	8.10 (0.20)	7.15 (0.18)	7.95 (0.22)	8.31 (0.23)	6.90 (0.13)	7.74 (0.17)	8.10 (0.19)
Self-support	7.42 (0.14)	7.72 (0.16)	8.26 (0.19)	7.59 (0.18)	7.96 (0.21)	8.17 (0.22)	7.45 (0.13)	8.35 (0.16)	8.51 (0.51)
Insomnia	11.26 (0.32)	11.08 (0.34)	10.03 (0.40)	11.71 (0.39)	9.97 (0.45)	9.59 (0.47)	11.83 (0.29)	9.61 (0.34)	8.76 (0.38)
Alcohol use	4.06 (0.24)	3.82 (0.25)	3.41 (0.27)	3.04 (0.30)	2.77 (0.32)	2.88 (0.32)	3.48 (0.21)	2.93 (0.23)	3.03 (0.25)

**Table 2:** Linear mixed-effects models adjustment indices.

	AIC	BIC	Log-Likelihood	R^2^ Marginal	R^2^ Conditional	ICC
Psychological Distress	14,917.21	14,970.53	−7443.40	0.0611	0.6570	0.635
Depression	11,426.50	11,495.31	−5705.79	0.0444	0.6347	0.618
Anxiety	10,873.79	10,945.08	−5430.68	0.0357	0.6412	0.628
Stress	11,047.79	11,118.08	−5517.18	0.0638	0.5661	0.537
Insomnia	12,250.95	12,316.08	−6116.17	0.0307	0.6253	0.613
Alcohol use	10,161.33	10,236.84	−5076.56	0.0097	0.8575	0.856
Well-being	17,705.63	17,743.99	−8830.30	0.0180	0.7714	0.767
Positive Emotion	11,613.72	11,679.90	−5798.26	0.0181	0.7443	0.740
Engagement	11,693.77	11,759.17	−5837.89	0.0112	0.6269	0.623
Relationships	12,044.47	12,108.58	−6012.59	0.0061	0.7176	0.716
Meaning	12,312.13	12,375.15	−6145.88	0.0132	0.7533	0.750
Accomplishment	11,465.47	11,532.17	−5724.39	0.0251	0.7071	0.700
Health	12,000.53	12,064.87	−5990.74	0.0069	0.7257	0.724
Negative Emotion	11,488.16	11,554.71	−5735.66	0.0346	0.6989	0.688
Emotion Regulation Skills	16,022.99	16,068.51	−7992.56	0.0588	0.6404	0.618
Awareness	8554.22	8633.81	−4275.21	0.0372	0.5570	0.540
Modification	8655.54	8734.62	−4325.61	0.0330	0.5343	0.518
Understanding	8924.01	9001.94	−4459.28	0.0358	0.5723	0.556
Tolerance	9075.27	9152.45	−4534.53	0.0391	0.5530	0.535
Acceptance	8682.49	8761.36	−4338.99	0.0351	0.4999	0.482
Clarity	8925.00	9003.06	−4459.83	0.0343	0.6236	0.610
Bodily Sensations	9230.98	9307.32	−4611.96	0.0261	0.4847	0.471
Confrontation	9104.52	9181.31	−4548.96	0.0391	0.4217	0.398
Self-Support	8884.07	8962.06	−4439.33	0.0236	0.5165	0.505

**Note.** All linear mixed-effects models were estimated using Restricted Maximum Likelihood (REML).

AIC = Akaike Information Criterion; BIC = Bayesian Information Criterion; LogLik = Log-likelihood. Marginal R^2^ reflects the variance explained by fixed effects alone; Conditional R^2^ reflects the variance explained by both fixed and random effects. ICC = Intraclass Correlation Coefficient, indicating the proportion of total variance attributable to between-participant differences.

**Table 3:** Results from linear mixed-effects models for all outcomes.

	Time					Group					Time * Group				
	ddl1 (num.)	dd2 (den.)	F	P	η^2^p	ddl1 (num.)	dd2 (den.)	F	p	η^2^p	ddl1 (num.)	dd2 (den.)	F	p	η^2^p
Psychological distress	2	1156	92.9	<0.001	0.14	2	955	10.1	<0.001	0.02	4	1157	12	**<0.001**	0.04
Depression	2	1160	70.72	<0.001	0.11	2	952	5.91	0.003	0.01	4	1161	5.71	**<0.001**	0.02
Anxiety	2	1160	47.89	<0.001	0.08	2	956	6.66	<0.001	0.01	4	1161	7.5	**<0.001**	0.03
Stress	2	1202	74.7	<0.001	0.11	2	957	11.6	<0.001	0.02	4	1203	12.3	**<0.001**	0.04
Well-being	2	1083	45.95	<0.001	0.08	2	956	0.85	0.427	0.00	4	1084	3.94	**0.004**	0.01
Positive emotions	2	1088	42.36	<0.001	0.07	2	953	0.45	0.638	0.00	4	1089	4.64	**0.001**	0.01
Engagement	2	1126	13.42	<0.001	0.02	2	952	1.61	0.200	0.00	4	1127	2.16	0.072	0.01
Relationships	2	1104	12.14	<0.001	0.02	2	962	0.44	0.645	0.00	4	1105	0.72	0.577	0.00
Meaning	2	1089	25.90	<0.001	0.04	2	957	1.26	0.283	0.00	4	1089	2.87	**0.022**	0.01
Accomplishment	2	1099	58.35	<0.001	0.10	2	952	0.58	0.562	0.00	4	1100	3.10	**0.015**	0.01
Health	2	1107	13.51	<0.001	0.02	2	968	0.32	0.723	0.00	4	1108	2.46	**0.044**	0.01
Loneliness	2	1155	5.49	0.004	0.01	2	958	1.35	0.260	0.00	4	1156	3.66	**0.006**	0.01
Negative emotions	2	1114	52.12	<0.001	0.09	2	964	6.44	0.002	0.01	4	1115	8.01	**<0.001**	0.03
Emotion regulation	2	1134	112.82	<0.001	0.17	2	957	1.54	0.215	0.00	4	1135	7.69	**<0.001**	0.03
Emotional awareness	2	1160	56.80	<0.001	0.09	2	949	1.03	0.358	0.00	4	1161	7.04	**<0.001**	0.02
Modification	2	1173	37.05	<0.001	0.06	2	951	4.68	0.009	0.01	4	1174	4.20	**0.002**	0.01
Understanding	2	1134	59.53	<0.001	0.09	2	931	0.03	0.966	0.00	4	1136	6.35	**<0.001**	0.02
Tolerance	2	1159	56.23	<0.001	0.09	2	945	1.96	0.142	0.00	4	1160	4.95	**<0.001**	0.02
Acceptance	2	1195	45.14	<0.001	0.07	2	954	2.20	0.111	0.00	4	1196	4.21	**0.002**	0.01
Clarity	2	1129	67.19	<0.001	0.11	2	950	0.23	0.792	0.00	4	1130	3.59	**0.006**	0.01
Sensation	2	1199	36.96	<0.001	0.06	2	952	0.19	0.830	0.00	4	1201	2.28	**0.059**	0.01
Confrontation	2	1198	47.46	<0.001	0.07	2	905	2.44	0.088	0.00	4	1199	1.87	0.114	0.00
Self-support	2	1190	29.48	<0.001	0.05	2	961	1.58	0.21	0.00	4	1191	2.40	**0.048**	0.01
Insomnia	2	1190	51.29	<0.001	0.08	2	981	1.67	0.19	0.00	4	1191	6.64	**<0.001**	0.02
Alcohol use	2	1104	10.66	<0.001	0.02	2	966	3.14	0.04	0.00	4	1104	1.82	0.120	0.01

**Note.** All results are from linear mixed-effects models with repeated measures nested within participants. *η*^*2*^*p* = partial eta squared. T = Time; G = Group; Int = Time × Group. Bold values indicate statistically significant interaction effects (*p* < .05).

Post hoc comparisons showed that both the hybrid and online-only groups experienced greater reductions in total psychological distress, depressive, anxiety, and stress symptoms (**Tables S2**) than the control group at post-intervention (T2) and follow-up (T3), with no significant differences between the two intervention formats at any time point. Effect sizes for the interactions were small to moderate (η^2^p = 0.02–0.04), suggesting clinically meaningful but gradual improvements over time.

##### 3.3.1.2 Well-being

Significant main effects of time were observed for overall well-being (PERMAH total) and most PERMAH dimensions, including positive emotions, engagement, relationships, meaning, accomplishment, health, loneliness, and negative emotions, indicating global improvements across the study period (all *ps* < 0.01). No significant main effects of group were found for most well-being outcomes, suggesting comparable average levels across conditions when collapsing across time.

However, significant time × group interactions were observed for overall well-being (PERMAH total) as well as for several PERMAH dimensions, including positive emotions, meaning, accomplishment, health, loneliness, and negative emotions (all *ps* ≤ 0.044). These interactions indicate differential change trajectories across groups, with more pronounced improvements over time in the intervention conditions.

Post hoc analyses revealed that reductions in negative emotions were particularly marked in the online-only group compared to the control group at both post-intervention and follow-up, while differences between the hybrid and online-only groups were not significant (**Tables S3-S4**). No other PERMAH dimensions showed consistent between-group differences after correction, supporting the interpretation of broadly shared gains in well-being with some modality-specific advantages.

#### 3.3.2 Effects on behavioural outcomes

For insomnia severity (ISI), a significant main effect of time and a significant time × group interaction were observed (ps < 0.001), indicating greater reductions in insomnia symptoms in the intervention groups compared to the control group over time. Post hoc contrasts showed no statistically significant between-group differences at individual time points after correction, suggesting that the interaction reflected differential trajectories rather than discrete cross-sectional differences (**Table S5**).

For alcohol use (AUDIT), a significant main effect of time was found (*p* < .001), along with a small but significant main effect of group (*p* = .040). The time × group interaction was not significant, indicating similar temporal patterns of change across conditions.

#### 3.3.3 Effects of the Intervention on emotion regulation skills

For overall emotion regulation, a strong main effect of time was observed, *F*(2, 1134) = 112.82, *p* < .001, η^2^p = 0.17, indicating substantial improvements in emotion regulation skills across the study period. No significant main effect of group was found, *F*(2, 957) = 1.54, *p* = .215, but the time × group interaction was significant, *F*(4, 1135) = 7.69, *p* < .001, η^2^p = 0.03, suggesting differential patterns of skill acquisition across conditions. Post hoc comparisons on the ERSQ total score (**Table S6**) showed that, at post-intervention (T2), both intervention groups demonstrated significantly greater improvements in emotion regulation skills compared to the control group.

At the subscale level, significant time × group interactions were observed for several ERSQ dimensions, including emotional awareness, modification, understanding, tolerance, acceptance, clarity, and self-support (all *ps* ≤ 0.048). Post hoc analyses (**Table S7**) indicated that the online-only group showed greater improvements in emotion modification skills, whereas the hybrid group exhibited more pronounced gains in emotion tolerance and acceptance, particularly at post-intervention (Supplementary Material). No sustained between-group differences were observed at follow-up for most subscales, indicating convergence of emotion regulation skills over time.

### 3.4 Clinical change trajectories

Exploratory multinomial logistic regressions and chi-square tests were conducted to examine clinical transitions (aggravation, improvement, remission, or stability) across psychological distress (DASS-21), insomnia (ISI), and alcohol use (AUDIT). Because these analyses involved multiple outcomes, time points, and contrasts, they should be interpreted cautiously. For psychological distress from T1 to T2, the online-only group had lower odds of aggravation than the control group (OR = 0.49, 95% CI [0.26, 0.90], *p* = .022); this difference was not maintained at T3 (OR = 0.75, 95% CI [0.37, 1.54], *p* = .437). Complementary descriptive tables from the chi-square analyses showed 9 observed cases of aggravation in the online-only group and 33 in the control group at T2. At T2, the hybrid group had higher odds of improvement than the control group (OR = 3.75, 95% CI [1.22, 11.52], *p* = .021), but this difference was not maintained at T3. The complementary chi-square table showed 27 observed improvement cases in the hybrid group and 21 in the control group. Both intervention groups had higher odds of remission from psychological distress than the control group at T2 (hybrid: OR = 3.07, 95% CI [1.39, 6.79], *p* = .006; online-only: OR = 2.68, 95% CI [1.42, 5.06], *p* = .002) and T3 (hybrid: OR = 3.53, 95% CI [1.44, 8.65], *p* = .006; online-only: OR = 3.78, 95% CI [1.71, 8.32], *p* < .001). In the complementary chi-square tables, the observed remission counts were 24, 36, and 13 at T2, and 26, 35, and 16 at T3, in the hybrid, online-only, and control groups, respectively. The intervention formats did not differ significantly in remission odds.

For insomnia, both intervention groups had higher odds of remission than the control group at T2 (hybrid: OR = 2.04, 95% CI [1.05, 3.93], *p* = .034; online-only: OR = 2.76, 95% CI [1.53, 4.98], *p* < .001) and T3 (hybrid: OR = 2.06, 95% CI [1.04, 4.08], *p* = .039; online-only: OR = 2.49, 95% CI [1.33, 4.68], *p* = .004). The online-only group also had higher odds of short-term insomnia improvement than the control (OR = 2.04, 95% CI [1.04, 4.01], *p* = .039) and hybrid groups (OR = 2.26, 95% CI [1.02, 4.98], *p* = .044), but these differences were not maintained at T3. No significant group differences in insomnia aggravation were observed, and no consistent intervention-related transition pattern emerged for alcohol use; several alcohol-related estimates showed signs of instability related to small cell sizes in some transition categories (see Supplementary File S2 for full results and cell counts). Baseline symptom severity emerged as a strong predictor of clinical change across all outcomes. Detailed clinical transition analyses, including cell sizes and full model outputs, are provided in Supplementary File S2.

### 3.5 Role of program completion in outcome trajectories

Sensitivity analyses including program completion as a continuous covariate are reported in Supplementary File S3. Results showed that engagement had a limited but outcome-specific influence on intervention effects. Higher completion was associated with lower depressive symptoms, fewer sleep difficulties, and higher global well-being, as well as with selected well-being dimensions (relationships, meaning, accomplishment). In contrast, completion was not significantly related to anxiety, stress, alcohol use, loneliness, or negative emotions.

For emotion regulation, completion did not predict overall ERSQ scores, with the exception of confrontation of situations, suggesting that some regulatory skills may be more sensitive to repeated exposure than others. Importantly, Time × Group interactions remained significant for most outcomes after controlling for completion, indicating that group-related differences in change trajectories were not explained by engagement level alone. This suggests that the effectiveness of the intervention formats reflects both structural characteristics of delivery and, to a lesser extent, individual engagement.

## 4 Discussion

This randomized controlled trial evaluated the efficacy of the ETUCARE digital mental health intervention, comparing its online-only and hybrid formats with a control group. Overall, findings support the effectiveness of both intervention modalities in improving mental health outcomes among university students, with distinct trajectories across formats and symptom profiles.

### 4.1 Effectiveness of the ETUCARE intervention

Both intervention groups showed more favorable trajectories in psychological distress than the control group, with small but consistent time × group interaction effects (η^2^p = 0.02–0.04 across total distress, depression, anxiety, and stress). These findings align with previous research indicating the efficacy of DMHIs in reducing depression, anxiety, and stress symptoms (Lattie et al., 2019; Harrer et al., 2019). No significant differences emerged between the online and hybrid formats, suggesting comparable efficacy. The absence of significant differences between the online-only and hybrid formats suggests that, in this context, both delivery modalities were comparably effective in reducing psychological distress. This finding may seem at odds with prior research indicating that guided digital interventions generally outperform self-guided formats (Grist et al., 2019; Mohr et al., 2011). However, the magnitude and consistency of these effects vary across populations and study designs (Gan et al., 2021); supporting prior research showing equivalent effectiveness of guided and unguided formats (Madrid-Cagigal et al., 2025). Exploratory clinical transition analyses (Supplementary File S2) were broadly consistent with the mixed-model findings, suggesting that both ETUCARE formats increased the likelihood of clinically meaningful change, particularly remission, for psychological distress and insomnia. The hybrid format showed a relative advantage for symptom improvement, whereas the online-only format was associated with a lower likelihood of symptom aggravation at post-test, an effect that was not maintained at follow-up. However, given the exploratory nature of these analyses and the limited persistence of the aggravation-related finding, this result should not be interpreted as evidence that online-only delivery actively protects against clinical worsening; rather, it may reflect a shorter-term, format-specific difference that warrants replication. The present study assessed only symptom deterioration and did not systematically capture other potential negative or unwanted effects attributable to the intervention. Future trials should combine repeated validated outcome measures with direct participant reports of adverse events, novel symptoms, and reasons for dropout, while distinguishing events associated with the intervention from those arising from external circumstances (Rozental et al., 2014). Baseline symptom severity was, independently, a strong predictor of remission across all groups. While this finding does not by itself establish differential efficacy by severity level, it is broadly consistent with the wider literature on stepped-care models, which posit that scalable, low-intensity digital formats can serve as first-line support, with more structured, guided approaches reserved for individuals with higher clinical needs (Lattie et al., 2019; van Straten et al., 2015). Guided interventions may be especially beneficial in more clinical profiles, while self-guided formats may be sufficient in preventive or lower-severity contexts (Lipschitz et al., 2023; Moshe et al., 2021).

The inclusion of well-being outcomes based on the PERMAH framework allowed for a broader assessment of ETUCARE's impact beyond symptom reduction. According to Seligman (Seligman, 2011), flourishing reflects the presence of core psychological capacities (positive emotions, engagement, relationships, meaning, accomplishment, and health) that support resilience and adaptive functioning. In the present trial, small but significant improvements were observed across several PERMAH dimensions (η^2^p = 0.009–0.028), with the online-only format showing more consistent advantages across subscales and in particularly reductions in negative emotions. These effects align with meta-analytic evidence indicating modest but reliable improvements in student well-being following DMHIs (Ferrari et al., 2022) and with the small-to-moderate effect observed here for overall well-being (η^2^p = 0.014), supporting prior findings that self-guided digital programs can enhance positive psychological resources alongside symptom outcomes (Krifa et al., 2022). These findings are particularly relevant in prevention-oriented contexts, where strengthening protective factors is central. In line with Keyes' concept of “complete mental health” (Keyes, 2002), ETUCARE contributes to public health efforts to promote positive functioning and long-term well-being in young adults through scalable digital strategies (Patel et al., 2007; World Health Organization, 2022; Huen et al., 2016; Litvin et al., 2023).

ETUCARE was associated with significant improvements in sleep difficulties over time with a small-to-moderate effect size (*η*^*2*^*p* = 0.022). Although no corrected between-group differences emerged, the online-only group showed descriptively larger within-group from T1 to T3. These findings are consistent with prior literature showing that self-guided, flexible digital interventions can yield strong adherence and meaningful improvements in sleep-related outcomes, even without therapist involvement (van Straten et al., 2018; Zachariae et al., 2016). Digital CBT-I approaches, particularly when modular and self-paced, may enhance engagement and application of behavioural strategies such as sleep hygiene and stimulus control (Luik et al., 2017).

In contrast, changes in alcohol use were modest. Mixed-effects models revealed a small overall effect of time but no significant group differences, indicating uniform reductions across conditions. Only the online-only group showed a short-term decrease that was not sustained at follow-up. These results align with prior studies showing limited effects of non-targeted digital interventions on substance use unless specific motivational or normative components are included (Kaner et al., 2018; Riper et al., 2014).

Overall, these findings indicate that sleep-related behaviours are more responsive to general well-being interventions, particularly in flexible self-guided formats, whereas reductions in risky alcohol use may require more targeted, motivationally anchored, and potentially personalized intervention components, especially for students with elevated baseline risk.

The ETUCARE intervention produced significant improvements in global emotion regulation skills over time (η^2^p = 0.026), with both the online-only and hybrid formats outperforming the control group at post-test. The absence of differences between formats on the total ERSQ score suggests comparable efficacy in strengthening overall regulation capacities, consistent with prior evidence showing that self-paced digital interventions can enhance emotion regulation skills, which are recognized mediators of psychological well-being and protective factors against mental health deterioration (Berking and Lukas, 2015; Domhardt et al., 2021).

Subscale analyses revealed small but meaningful format-specific patterns. The hybrid format showed advantages in Acceptance and Tolerance, likely reflecting the supportive interpersonal context of group-based workshops, which can foster normalization, shared vulnerability, and nonjudgmental emotional processing (Zaki and Williams, 2013; Moore et al., 2022). In contrast, the online-only format yielded stronger effects on Emotion Modification aligning with the emphasis of digital programs on autonomous coping strategies such as cognitive reframing and behavioural activation (Domhardt et al., 2021).

Together, these findings suggest complementary mechanisms across delivery formats: hybrid interventions may preferentially support acceptance-based regulation, whereas online formats may strengthen change-oriented and self-directed skills. Given that emotion regulation deficits are transdiagnostic risk and protective factors for anxiety, depression, and stress (Aldao et al., 2010; Aldao and Dixon-Gordon, 2014), these improvements likely contributed to the broader clinical and well-being gains observed. Future research should examine whether specific regulation skills (e.g., emotion modification versus acceptance-based processes) mediate intervention effects, as suggested by prior work highlighting their distinct and sometimes independent roles in mental health outcomes (Berking et al., 2012).

### 4.2 Engagement and attrition

Results indicated strong initial engagement, followed by substantial attrition in research follow-up, particularly in the online-only group. This pattern is consistent with prior evidence showing that unguided DMHIs often achieve high early uptake but experience elevated attrition, especially in the absence of human support or incentives (Linardon et al., 2019). Higher retention in the hybrid group may reflect the anchoring effect of in-person sessions, which provide social structure and accountability, in line with findings highlighting human contact as a key determinant of sustained engagement in digital health interventions (Baumel and Kane, 2018). Importantly, attrition primarily reflected non-completion of follow-up assessments rather than disengagement from the intervention itself: among participants who accessed the platform, over 70% completed all eight modules, with no difference between online-only and hybrid formats. This underscores the need to distinguish adherence to intervention content from participation in research assessments.

Notably, adherence levels were high relative to those typically reported in youth DMHIs, where completion rates often range from 13% to 60% (Linardon et al., 2019; Potts et al., 2025). This may reflect strong alignment between ETUCARE's design and students' needs, including thematic relevance, interactivity, and accessible language that are key drivers of engagement in digital interventions (Potts et al., 2025; Perski et al., 2017). The absence of format differences in module completion suggests that content quality alone was sufficient to sustain engagement, contrasting with prior work favoring guided formats (Lipschitz et al., 2023). The gap between intervention adherence and research participation likely reflects pragmatic barriers, including lack of incentives, separation between the intervention platform and survey tools, academic constraints, and limited perceived value of questionnaires. Future studies should therefore prioritize integrated assessments, personalized reminders, user feedback, and light non-monetary incentives to improve longitudinal data completeness.

Sensitivity analyses including program completion as a continuous covariate (Supplementary File S3) showed that engagement contributed modestly but meaningfully to several outcomes, without accounting for group-related differences in change trajectories. Completion was associated with improvements in depressive symptoms, insomnia, and selected well-being dimensions (meaning, accomplishment, relationships), consistent with a dose-related effect for these outcomes. In contrast, completion was not significantly related to anxiety, stress, alcohol use, or most emotion regulation skills, except for confrontation of situations, suggesting domain-specific sensitivity to cumulative exposure. Importantly, significant Time × Group interactions persisted after controlling for completion, indicating that group differences cannot be reduced to quantitative exposure alone but likely reflect qualitative and contextual features of intervention delivery, including access to structured content, intervention framing, and the presence of human support in the hybrid condition. Completion and intervention format thus appear to exert complementary rather than redundant influences on outcomes. However, completion was operationalized as a global percentage of modules completed, which may insufficiently capture qualitative aspects of engagement (e.g., depth of reflection or adherence to exercises). Future studies should incorporate more fine-grained engagement indicators (e.g., time spent, activity-level engagement, perceived usefulness) to better characterize mechanisms of change (Choi et al., 2023).

### 4.3 Strengths, limitations, and future directions

This study benefits from a large, multi-regional sample recruited across six universities in France, enhancing the generalizability of findings across diverse academic and regional contexts. The distribution of academic levels aligns with ETUCARE's preventive focus on early stages of higher education. The overrepresentation of female participants reflects well-documented patterns in mental health research and related academic fields, likely driven by both higher prevalence of internalizing symptoms and greater research engagement among women (Seedat et al., 2009).

Several limitations warrant consideration. First, reliance on self-report measures may introduce reporting biases and the absence of objective behavioural indicators limits the robustness of conclusions regarding observed changes. Second, the follow-up period was relatively short, restricting conclusions about long-term maintenance of benefits. Third, recruitment within shared institutional settings raises the possibility of cross-condition contamination. Fourth, recruitment and intervention delivery occurred across four waves covering different periods of the academic year. Although participants within each wave followed a common schedule and randomization helped distribute temporal influences across conditions, variations in academic workload, teaching schedules, and examination periods may have contributed to outcome variability; wave and academic-calendar effects were not explicitly modelled. Fifth, mediation analyses were not conducted, precluding firm conclusions regarding underlying mechanisms of change, such as emotion regulation or engagement. Finally, the uniform intervention dose did not allow for individual tailoring based on baseline severity. Future research should explore adaptive or stepped-care designs to optimize intervention intensity, effectiveness, and scalability.

This randomized controlled trial found small but statistically significant differential improvements in psychological distress, insomnia, emotion regulation skills, and selected well-being outcomes for ETUCARE relative to a waitlist control condition. The online-only and hybrid formats produced broadly comparable global outcomes, offering flexible yet impactful options for student mental health services. Decision-makers can choose delivery mode based on resources, severity profiles, and user preference. The online format may be ideal for broad, scalable interventions, while blended models may offer additional value for students with more complex needs, consistent with stepped-care principles. Moreover, embedding digital tools within campus health ecosystem, rather than offering them as stand-alone intervention, may increase uptake, visibility, and impact.

## Funding

This research was supported by the French Institute for Public 10.13039/100005622Health Research (IReSP). The design and initial development of the ETUCARE program were funded by the Regional Health Agency of Bourgogne-Franche-Comté (ARS Bourgogne-Franche-Comté).

## Declaration of competing interest

The authors declare that they have no known competing financial interests or personal relationships that could have appeared to influence the work reported in this paper.

## Appendix A Supplementary data

Supplementary File S1 Full description of the ETUCARE program

Supplementary File S2 – Clinical Transition Models and Tables

Supplementary File S3 Sensitivity analyses including program completion

Table S1 Baseline equivalence across study groups on primary and secondary outcomes

Supplementary Tables S2-S7 Post hoc analyses and estimated marginal mean differences
