# Supplementary material for: Efficacy of a digital mental health program for university students: A randomized controlled trial comparing online and hybrid delivery
Source: Internet Interv. 2026 Sep 26;46:101002. doi: 10.1016/j.invent.2026.101002 (PMC13638797; doi:10.1016/j.invent.2026.101002)
Supplement: Supplementary file 1 [file mmc1.docx]

**Supplementary File S1.** Full description of the ETUCARE program

**Description of the digital ETUCARE program**

ETUCARE is an 8-week digital mental health intervention developed for university students in response to challenges associated with the COVID-19 pandemic. The program was created using a participatory co-design approach involving students and multidisciplinary experts. Initially, students’ mental health needs, expectations, and preferred digital features were explored through an online survey and qualitative interviews (unpublished data). These insights informed the overall structure and content of the program. Subsequently, three master’s students in public health, psychology, and management–marketing contributed to the iterative development process by testing early versions of the modules and providing feedback to enhance usability, relevance, and engagement.

The final content was developed collaboratively by a multidisciplinary team including a full professor, specialists in clinical psychology, cognitive-behavioral therapy, emotion regulation in young people, mindfulness-based and positive psychology interventions, as well as public health professionals with expertise in prevention and participatory program design. ETUCARE integrates evidence-based strategies drawn from cognitive-behavioral therapy, positive psychology, mindfulness, and lifestyle medicine. The program comprises eight asynchronous e-learning modules addressing key domains of student mental health: mental health literacy, stress management, procrastination and academic motivation, sleep and insomnia, self-awareness, emotion regulation, interpersonal relationships, and a concluding booster module (**Table S1.1**).

Each module lasted approximately 45 minutes and combined psychoeducational content with short videos, testimonials reflecting student experiences, interactive tools, quizzes (e.g., sleep habits, stressor identification), and weekly practice exercises. Exercises were designed to be brief (5–15 minutes) to facilitate integration into students’ academic routines. The intervention was delivered entirely online, without face-to-face contact; participants received automated email notifications and reminders throughout the program. The most recent version of ETUCARE is accessible via https://elearning.promotion-sante-bfc.org/ (accessed January 21, 2026).

**Table S1.1. Overview of the ETUCARE digital program**

| **Module** | **Core content** | **Weekly practices and activities** |
| --- | --- | --- |
| **Module 1: Mental Health Literacy** | Conceptualization of mental health and positive mental health. Overview of common psychological difficulties in students. Risk and protective factors for mental health. Available support resources and help-seeking pathways. Introduction to the ETUCARE program structure and timeline | Online self-assessment questionnaires related to mental health |
| **Module 2: Self-Awareness and Personal Resources** | Introduction to self-esteem and self-concept. Identification and use of personal strengths. Clarification of personal values and meaningful life directions | VIA Character Strengths assessment. Strengths exercises. Vision board creation. Engagement in value-based actions |
| **Module 3: Procrastination and Academic Motivation** | Conceptual framework of procrastination in daily and academic life. Identification of individual procrastination triggers. Self-assessment of procrastination behaviors. Role of self-criticism, perfectionism, and fear of failure. Key motivational processes involved in learning | Self-compassion exercise. Mindful breathing practice. SMART goal-setting |
| **Module 4: Stress Management** | Definition of stress and stressors. Physiological and emotional consequences of stress. Role of physical activity and exposure to nature in stress reduction. Introduction to evidence-based relaxation strategies | Body scan meditation. Controlled breathing exercises. Progressive muscle relaxation. Scheduling restorative activities. Increasing physical activity. Enhancing contact with natural environments. Problem-solving strategies |
| **Module 5: Emotion Regulation** | Functional role of emotions in daily life. Cognitive–behavioral links between thoughts, emotions, and behaviors. Identification of cognitive distortions and maladaptive thinking patterns. Monitoring mood. Association between lifestyle behaviors and emotional states. Introduction to positive emotions and strategies to enhance them | Cognitive reappraisal exercises. Distress tolerance strategies. Attentional refocusing toward positive experiences. Gratitude wall. Savoring exercises |
| **Module 6: Interpersonal Relationships** | Importance of supportive and meaningful social relationships. Development of communication and assertiveness skills. Managing interpersonal difficulties and social challenges | Assertiveness self-assessment. Random acts of kindness. Focusing on positive relational experiences. Active listening practice. Assertiveness training exercises. Gratitude letter |
| **Module 7: Sleep and Insomnia** | Basic principles of sleep and insomnia. Cognitive–behavioral model linking thoughts, emotions, and behaviors. Interactions between sleep, physical activity, and diet. Assessment of sleep quality and sleep hygiene | Mindfulness-based sleep meditation. Worry journal. Sleep hygiene strategies. Stimulus control techniques |
|  |  |  |
| **Module 8: Booster Session** | Review and consolidation of key concepts and skills introduced throughout the program | Continued practice of personally relevant exercises |

**Description of face-to-face workshops structure and content**

The ETUCARE program included eight standardized, weekly, 2-hour face-to-face group workshops delivered by trained facilitators, guided by a detailed pedagogical manual. Each workshop followed a consistent structure comprising: (1) an opening phase to foster group cohesion and reflective engagement (e.g., experiential exercises, mindfulness, or movement-based activities); (2) a psychoeducational component grounded in evidence-based psychology and neuroscience; (3) experiential and interactive exercises allowing participants to explore, practice, and personalize psychological skills; and (4) a closing phase focused on reflection, integration, and the presentation of between-session practices. Across the eight workshops, participants worked on core psychological domains relevant to student mental health, including mental health literacy and personal resources, self-knowledge and strengths, procrastination and goal-directed behavior, stress regulation, emotion understanding and regulation, interpersonal relationships and communication skills, sleep hygiene, and long-term self-care planning. Between-session practices were systematically proposed to facilitate transfer to daily life and support behavior change. The face-to-face workshops were designed to complement the digital ETUCARE modules by deepening experiential learning, supporting motivation and engagement, and facilitating the transfer of digitally introduced skills into real-life contexts through guided practice, peer interaction, and facilitator support.
