# Supplementary material for: Efficacy of a digital mental health program for university students: A randomized controlled trial comparing online and hybrid delivery
Source: Internet Interv. 2026 Sep 26;46:101002. doi: 10.1016/j.invent.2026.101002 (PMC13638797; doi:10.1016/j.invent.2026.101002)
Supplement: Supplementary file 2 [file mmc2.docx]

**Supplementary File S2 – Clinical Transition Models and Tables**

This file contains detailed results of the multinomial logistic regression analyses and chi-square contingency table***s*** examining the clinical potential of the intervention using categorical transition analyses. For three key outcomes (psychological distress, insomnia, and alcohol use) we classified participants into four clinical trajectories between T1 and T2, and T1 and T3: (1) stable, (2) improvement, (3) remission, and (4) aggravation. Transitions were defined using validated clinical cutoffs. Multinomial logistic regression models were used to predict clinical changes based on intervention group and baseline symptom severity. Two model specifications allowed for pairwise group comparisons using different reference categories (control vs. hybrid). To complement this approach, chi-square tests of independence were conducted to determine whether the distribution of transition categories differed significantly across the three study arms. Standardized residuals were examined to identify over- or under-representation of specific transition outcomes within each group.

**Multinomial Logistic Regression Analyses of Clinical Transitions**

***Psychological Distress***

The multinomial logistic regression model examining clinical transitions on the DASS-21 between T1 and T2 showed acceptable fit (*McFadden’s pseudo-R²* = .368, *AIC* = 883), indicating moderate explanatory power. The multinomial logistic regression model used to examine clinical change on the DASS-21 between baseline (T1) and follow-up (T3) demonstrated good explanatory power (*McFadden’s pseudo-R²* = .382, *AIC* = 634). The reference category was “stable”, allowing estimation of the likelihood of symptom aggravation, improvement, or remission, as compared to no change in clinical severity. Multinomial logistic regression results predicting DASS-21 clinical transitions between T1 and T2 and between T1 and T3 are presented in **Table S2.1.**

**Aggravation vs. stable**

Participants in the online-only group had significantly lower odds of aggravating their symptoms compared to those in the control group (*β* = -0.717, *p* = .022) at T2. No significant difference was observed between the hybrid group and the control group (*β* = -0.265, *p* = .433), nor between the online and hybrid groups (*β* = -0.452, *p* = .241) for transitions form T1 to T2. No group effect on aggravation was observed for transitions from T1 to T3 (*p*s >.10)

Initial symptom severity at T1 strongly predicted aggravation: participants with mild (*β* = 1.73, *p* < .001) or moderate (*β* = 1.62, *p* < .001) symptoms were more likely to worsen than asymptomatic participants. Participants with mild initial symptom severity at T1 were also more likely to worsen their symptoms at T3 (*β* = 1.90, *p* < .001).

**Improvement vs. stable**

Participants in the hybrid group were significantly more likely to show symptom improvement than those in the control group (*β* = 1.32, *p* = .021). The difference between the online-only and control groups was not statistically significant (*β* = 0.71, *p* = .139), nor was the difference between the online-only and hybrid groups (*β* = -0.61, *p* = .303). No significant effect of the intervention formats on symptom improvement was observed at T3 (all *ps*>.10). Baseline symptom severity was not a significant predictor of improvement in these models (all *ps* >10).

**Remission vs. stable**

Participants in the hybrid group (*β* = 1.12, *p* = .006) and in the online group (*β* = 0.99, *p* = .002) were significantly more likely to reach clinical remission than those in the control group. No significant difference was found between the hybrid and online-only formats (*β* = -0.13, *p* = .752). Participants in the online (*β* = 1.329, *p* < .001) and hybrid groups (*β* = 1.262, *p* = .006) were significantly more likely to reach remission than controls at T3. As expected, initial symptom severity was a strong predictor of remission, with participants showing higher baseline symptoms being more likely to reach asymptomatic status at T2 (*p* < .001 for all DASS-T1 levels ≥1 compared to level 0). Again, baseline severity was a strong positive predictor of remission at T3 again (*p* < .001 for all DASS-T1 levels ≥1 compared to level 0).

**Table S2.1. Multinomial Logistic Regression Predicting DASS-21 Clinical Transitions From Baseline to T2 and T3**

| Transition | Predictor | *β (SE)* | *Z* | *p* | Model |
| --- | --- | --- | --- | --- | --- |
| Aggravation T1-T2 | O – C | -0.717 (0.314) | -2.28 | .022 | M1 |
|  | H – C | -0.265 (0.338) | -0.78 | .433 | M1 |
|  | O – H | -0.452 (0.385) | -1.17 | .241 | M2 |
| Improvement T1-T2 | H – C | 1.321 (0.573) | 2.30 | .021 | M1 |
|  | O – C | 0.714 (0.482) | 1.48 | .139 | M1 |
|  | O – H | -0.607 (0.589) | -1.03 | .303 | M2 |
| Remission T1-T2 | H – C | 1.121 (0.405) | 2.77 | .006 | M1 |
|  | O – C | 0.987 (0.324) | 3.05 | .002 | M1 |
|  | O – H | -0.134 (0.424) | -0.32 | .752 | M2 |
| Aggravation T1-T3 | O – C | -0.282 (0.363) | -0.78 | .437 | M1 |
|  | H – C | -0.200 (0.390) | -0.51 | .608 | M1 |
|  | O – H | -0.083 (0.413) | -0.20 | .841 | M2 |
| Improvement T1-T3 | H – C | 0.837 (0.632) | 1.32 | .186 | M1 |
|  | O – C | 0.375 (0.558) | 0.67 | .501 | M1 |
|  | O – H | -0.461 (0.650) | -0.71 | .478 | M2 |
| Remission T1-T3 | H– C | 1.262 (0.457) | 2.76 | .006 | M1 |
|  | O – C | 1.329 (0.403) | 3.30 | <.001 | M1 |
|  | O – H | 0.068 (0.463) | 0.15 | .883 | M2 |

**Note.** M1 = Model 1 (control group as reference); M2 = Model 2 (hybrid group as reference); O = Online-only group; H= Hybrid group; C = Control group. DASS-T1 levels refer to baseline severity (0 = asymptomatic, 1 = mild, 2 = moderate, 3 = severe).

***Sleep problems***

The multinomial logistic regression models examining transitions in insomnia severity (ISI) showed acceptable fit between T1 and T2 (*McFadden’s pseudo-R²* = .270, *AIC* = 1119) and between T1 and T3 (*McFadden’s pseudo-R²* = .262, *AIC* = 855), suggesting moderate explanatory capacity. Multinomial logistic regression results predicting ISI clinical transitions between T1 and T2 and between T1 and T3 are presented in **Table S2.2**.

**Aggravation vs. Stable**

No intervention group had a significantly higher or lower risk of aggravation than the control group (*ps* >.10) between T1 and T2 and no significant difference was observed between online vs hybrid groups (*p* = .322). Similar non-significant comparisons were observed for clinical transitions form T1 to T3 (*ps* >.10). Participants with higher insomnia severity at baseline, especially moderate (*β* = -1.873, *p* < .001) or severe (*β* = -22.00, *p* < .001) were significantly less likely to worsen at T2 and T3, suggesting ceiling effects or regression to the mean.

**Improvement vs. stable**

Participants in the online group were significantly more likely to improve their ISI category than those in the control group (*β* = +0.712, *p* = .039) and those in the hybrid group (*β* = 0.814, *p* = .044) from T1 to T2. These differences were not maintained at T3 (*p* >.50). No significant differences were observed between hybrid vs control for transitions to T2 (*β* = -0.103, *p* = .795) and to T3 (*β* = 0.221, *p* = .642). Students with moderate (β = 92.82, *p* < .001) to severe (*β* = 93.56, *p* < .001) symptoms at baseline were significantly more likely to improve over time (T3).

**Remission vs. stable**

Students in the online group (*β* = 1.014, *p* < .001) and in the hybrid group (*β* = 0.711, *p* = .034) were significantly more likely to remit from insomnia symptoms than controls at T2. Both online group (*β* = 0.914, *p* = .004) and hybrid (*β* = 0.722, *p* = .039) were effective in increasing the odds of full remission of insomnia by T3. No significant difference between the two intervention groups at T2 (*β* = 0.303, *p* = .341) and T3 (*β* = 0.192 *p* = .559). As expected, students with higher ISI scores at baseline were much more likely to achieve remission (*p* < .001 for all ISI-T1 levels ≥1 compared to level 0) for each transition.

**Table S2.2. Multinomial Logistic Regression Predicting ISI Clinical Transitions From Baseline to T2 and T3**

| Transition | Predictor | *β (SE)* | *Z* | *p* | Model |
| --- | --- | --- | --- | --- | --- |
| Aggravation T1-T2 | O – C | -0.246 (0.270) | -0.91 | .362 | M1 |
|  | H – C | 0.070 (0.296) | 0.24 | .813 | M1 |
|  | O – H | -0.316 (0.319) | -0.99 | .322 | M2 |
| Improvement T1-T2 | H – C | -0.103 (0.394) | -0.26 | .795 | M1 |
|  | O – C | 0.712 (0.345) | 2.06 | .039 | M1 |
|  | O – H | 0.814 (0.404) | 2.01 | .044 | M2 |
| Remission T1-T2 | H – C | 0.711 (0.336) | 2.12 | .034 | M1 |
|  | O – C | 1.014 (0.302) | 3.36 | <.001 | M1 |
|  | O – H | 0.303 (0.318) | 0.95 | .341 | M2 |
| Aggravation T1-T3 | O – C | -0.061 (0.336) | -0.18 | .855 | M1 |
|  | H – C | 0.436 (0.343) | 1.27 | .204 | M1 |
|  | O - H | -0.498 (0.359) | -1.39 | .165 | M2 |
| Improvement T1-T3 | H – C | 0.221 (0.475) | 0.47 | .642 | M1 |
|  | O – C | 0.292 (0.440) | 0.66 | .507 | M1 |
|  | O – H | 0.072 (0.453) | 0.16 | .874 | M2 |
| Remission T1-T3 | H – C | 0.722 (0.349) | 2.07 | .039 | M1 |
|  | O – C | 0.914 (0.321) | 2.84 | .004 | M1 |
|  | O – H | 0.192 (0.328) | 0.58 | .559 | M2 |

**Note.** M1 = Model 1 (control group as reference); M2 = Model 2 (hybrid group as reference); O = Online-only group; H= Hybrid group; C = Control group. DASS-T1 levels refer to baseline severity (0 = asymptomatic, 1 = mild, 2 = moderate, 3 = severe).

***Alcohol use***

The multinomial logistic regression models used to assess clinical transitions in alcohol consumption severity (AUDIT) demonstrated a strong fit (*McFadden’s pseudo-R²* = .455, *AIC* = 340) between T1 and T2 and between T1 and T3 (*McFadden’s pseudo-R²* = .505, *AIC* = 232). Multinomial logistic regression results predicting AUDIT clinical transitions between T1 and T2 and between T1 and T3 are presented in **Table S2.3**.

**Aggravation vs. Stable**

No significant difference between group in the likelihood of aggravation form T1 to T2 were observed (*ps* > .10). Marginal trend toward reduced aggravation from T1 to T3 (*β* = -2.136, *SE* = 1.162, *Z* = -1.84, *p* = .066) was observed between the hybrid vs. control. Significantly higher odds of aggravation were observed for students with moderate symptoms at baseline at T2 (*β* = 0.115, *p* < .001), and T3 (*β* = 3.72, *p* = .008).

**Improvement vs. Stable**

No significant differences were observed between online and control groups (*β* = 1.108, *SE* = 1.529, *Z* = 0.72, *p* = .469) and hybrid vs control (*β* = 5.970, *SE* = 19.80, *Z* = 0.30, *p* = .763). Participants in the online group were significantly less likely to improve compared to those in the hybrid group (*β* = -51.53, *p* < .001). This improvement was more likely among participants with elevated baseline alcohol use. Significantly higher odds of improvement were observed for students in the hybrid group with mild (*β* = 38.88, *p* = .023) moderate (*β* = 267.86, *p* < .001) and severe symptoms (*β* = +100.07, *p* < .001) at baseline. No significant between groups differences were observed for clinical transitions from T1 to T3 (all *ps* >.10).

**Remission vs. Stable**

For clinical transitions from T1 to T2, neither the online (*β* = 0.803, *p* = .144) nor the hybrid ETUCARE format (*β* = 0.646, *p* = .324) significantly increased remission from risky alcohol use compared to control. No significant difference was observed between online and hybrid (*β* = 0.157, *SE* = 0.701, *Z* = 0.22, *p* = .823). Regarding clinical transitions form T1 to T3, a marginal trend toward reduced remission was observed in the hybrid group compared to controls (*β* = -1.384, *p* = .069). No significant difference was observed between online and control (*β* = -0.357, *p* = .556) and between online and hybrid groups (*β* = 1.03, *p* = .214). All contrasts suggest greater likelihood of remission among those with initial alcohol risk (all *p* < .001).

**Table S2.3. Multinomial Logistic Regression Predicting AUDIT Clinical Transitions From Baseline to T2 and T3**

| Transition | Predictor | *β (SE)* | *Z* | *p* | Model |
| --- | --- | --- | --- | --- | --- |
| Aggravation T1-T2 | O – C | -0.358 (0.468) | -0.77 | .444 | M1 |
|  | H – C | 0.549  (0.589) | -0.93 | .352 | M1 |
|  | O – H | 0.190 (0.623) | 0.31 | .760 | M2 |
| Improvement T1-T2 | H – C | 5.970 (19.80) | 0.30 | .763 | M1 |
|  | O – C | 1.108 (1.529) | 0.72 | .469 | M1 |
|  | O – H | -51.53  (3.53) | -14.62 | <.001 | M2 |
| Remission T1-T2 | H – C | 0.646 (0.656) | 0.99 | .324 | M1 |
|  | O – C | 0.803 (0.550) | 1.46 | .144 | M1 |
|  | O – H | 0.157 (0.701) | 0.22 | .823 | M2 |
| Aggravation T1-T3 | O – C | -0.601 (0.622) | -0.97 | .334 | M1 |
|  | H – C | -2.136 (1.162) | -1.84 | .066 | M1 |
|  | O – H | 1.536 (1.161) | 1.32 | .186 | M2 |
| Improvement T1-T3 | H – C | -59.96 (136.69) | -0.44 | .661 | M1 |
|  | O – C | -59.93 (136.69) | -0.44 | .661 | M1 |
|  | O – H | 0.030 (1.576) | 0.02 | .985 | M2 |
| Remission T1-T3 | H – C | -1.384 (0.761) | -1.82 | .069 | M1 |
|  | O – C | -0.357 (0.608) | -0.59 | .556 | M1 |
|  | O – H | 1.026 (0.826) | 1.24 | .214 | M2 |

**Note.** M1 = Model 1 (control group as reference); M2 = Model 2 (hybrid group as reference); O = Online-only group; H= Hybrid group; C = Control group. DASS-T1 levels refer to baseline severity (0 = asymptomatic, 1 = mild, 2 = moderate, 3 = severe).

**Multinomial Logistic Regression Analyses: Cross-Time and Cross-Outcomes Synthesis**

For psychological distress, both intervention formats were associated with a significantly greater likelihood of remission relative to control at post-test and follow-up. The hybrid format was associated with significantly greater symptom improvement relative to control at post-test, whereas the online-only format was not. The online-only format was associated with lower odds of symptom aggravation relative to control at post-test only (*p* = .022); this effect was not maintained at follow-up (*p* = .437) and was not observed for insomnia or alcohol use. Initial symptom severity was a robust predictor of both deterioration and remission across time points, highlighting the heterogeneity of clinical trajectories among students with mild or moderate baseline distress.

The online-only format was associated with significantly greater short-term improvements in insomnia severity compared to both the control and hybrid formats, although these effects were not sustained at follow-up. Both intervention formats were associated with significantly greater odds of remission from insomnia relative to control at both T2 and T3. As expected, participants with higher baseline insomnia severity were the most likely to benefit from the intervention in terms of clinical remission, further underscoring the importance of initial symptom level in predicting outcomes.

For alcohol use, no significant differences relative to control were observed for improvement or remission at either T2 or T3. The direct comparison between the hybrid and online-only formats indicated significantly higher odds of improvement in the hybrid group at T2. No significant format differences were observed for improvement at T3. A marginal, non-significant trend toward reduced aggravation was observed in the hybrid group relative to control at follow-up (*p* = .066); no corresponding trend was observed for improvement at follow-up, for which all format contrasts were non-significant. Across models, baseline alcohol use severity was consistently associated with these transitions, a pattern also present in the control group and therefore reflecting the general predictive role of baseline severity for clinical trajectories rather than a specific effect of intervention format.

### **Group Differences in Transition Patterns**

To complement regression findings, chi-square tests of independence were conducted to examine associations between intervention group and clinical transitions (aggravation, improvement, remission, stability).
A significant association was found for psychological distress from T1 to T2, *χ²*(6) = 18.4, *p* = .005. The online-only group showed significantly fewer aggravation cases than expected (*z* = -2.32) and the control group significantly more (*z* = +2.67); the control group also showed significantly fewer remissions than expected (*z* = -2.21). The online-only group's higher-than-expected remission count did not reach the pre-specified significance threshold (*z* = 1.92) and is reported as a descriptive trend only. For insomnia severity, the association between intervention group and clinical transition from T1 to T2 was also significant, *χ²*(6) = 13.1, *p* = .042. The control group showed a significantly lower-than-expected number of remissions (*z* = -2.04); supporting the potential benefit of the intervention in sleep-related outcomes.

No statistically significant residuals were observed in alcohol use transitions, despite a significant global association from T1 to T3, χ²(6) = 13.0, *p* = .043; this global association could not be attributed to any specific group-transition combination. Full contingency tables, residuals, and observed vs. expected frequencies are reported in **Tables S2.4 to S2.9.** Taken together, these results indicate a consistent pattern of lower-than-expected aggravation and higher-than-expected remission in the control group for psychological distress and insomnia, but no comparable pattern for alcohol use.

**Chi-Square Tests of Independence on Clinical Transitions**

The chi-square test evaluates the overall association between intervention group and clinical transition type. The reported χ² and *p*-values refer to the global test of independence across all categories. Standardized residuals (z-scores) indicate cell-level deviations from expected frequencies. Residuals exceeding 1.96 are considered statistically significant and are shown in bold.

**Table S2.4 Clinical Transitions in Psychological Distress (DASS-21), T1–T2**

| Group | Aggravation  (Obs/Exp/z) | Improvement  (Obs/Exp/z) | Remission  (Obs/Exp/z) |
| --- | --- | --- | --- |
| Control | 33 / 17.4 / **+2.67** | 21 / 24.1 / -0.64 | 13 / 24.1 / **-2.21** |
| Hybrid | 18 / 20.5 / -0.54 | 27 / 26.5 / +0.10 | 24 / 24.0 / +0.00 |
| Online-only | 9 / 22.0 / **-2.32** | 28 / 25.4 / +0.50 | 36 / 24.9 / +1.92 |

*χ²*(6) = 18.4, *p* = .005

**Table S2.5. Clinical Transitions in Psychological Distress (DASS-21), T1–T3**

| Group | Aggravation  (Obs/Exp/z) | Improvement  (Obs/Exp/z) | Remission  (Obs/Exp/z) |
| --- | --- | --- | --- |
| Control | 25 / 18.4 / +1.50 | 19 / 21.6 / -0.56 | 16 / 27.1 / -1.86 |
| Hybrid | 18 / 19.2 / -0.36 | 24 / 21.8 / +0.47 | 26 / 22.8 / +0.67 |
| Online-only | 13 / 18.4 / -1.43 | 22 / 21.6 / +0.09 | 35 / 27.1 / +1.55 |

*χ²(*6) = 10.3, *p* = .114

**Table S2.6. Clinical Transitions in Insomnia (ISI), T1–T2**

| Group | Aggravation  (Obs/Exp/z) | Improvement  (Obs/Exp/z) | Remission  (Obs/Exp/z) |
| --- | --- | --- | --- |
| Control | 17 / 11.7 / +1.52 | 25 / 25.5 / -0.10 | 18 / 30.4 / **-2.04** |
| Hybrid | 11 / 9.6 / +0.45 | 24 / 25.6 / -0.31 | 25 / 20.8 / +0.89 |
| Online-only | 6 / 12.6 / -1.43 | 30 / 27.9 / +0.39 | 40 / 31.8 / +1.43 |

*χ²*(6) = 13.1, *p* = .042

**Table S2.7. Clinical Transitions in Insomnia (ISI), T1–T3**

| Group | Aggravation  (Obs/Exp/z) | Improvement  (Obs/Exp/z) | Remission  (Obs/Exp/z) |
| --- | --- | --- | --- |
| Control | 15 / 13.3 / +0.46 | 19 / 22.4 / -0.72 | 15 / 24.8 / -1.93 |
| Hybrid | 11 / 12.4 / -0.42 | 23 / 21.2 / +0.38 | 26 / 23.3 / +0.56 |
| Online-only | 8 / 13.3 / -1.43 | 24 / 22.4 / +0.35 | 32 / 24.8 / +1.43 |

*χ²(*6) = 9.89, *p* = .129.

**Table S2.8. Clinical Transitions in Alcohol Use (AUDIT), T1–T2**

| Group | Aggravation  (Obs/Exp/z) | Improvement  (Obs/Exp/z) | Remission  (Obs/Exp/z) |
| --- | --- | --- | --- |
| Control | 9 / 8.5 / +0.18 | 10 / 9.3 / +0.23 | 8 / 9.8 / -0.59 |
| Hybrid | 6 / 7.1 / -0.41 | 8 / 7.9 / +0.05 | 7 / 8.3 / -0.45 |
| Online-only | 7 / 8.4 / -0.42 | 11 / 9.2 / +0.58 | 12 / 9.7 / +0.75 |

*χ²(*6) = 1.55, *p* = .956

**Table S2.9. Clinical Transitions in Alcohol Use (AUDIT), T1–T3**

| Group | Aggravation  (Obs/Exp/z) | Improvement  (Obs/Exp/z) | Remission  (Obs/Exp/z) |
| --- | --- | --- | --- |
| Control | 15 / 13.3 / +0.46 | 19 / 22.4 / -0.72 | 15 / 24.8 / -1.93 |
| Hybrid | 11 / 12.4 / -0.42 | 23 / 21.2 / +0.38 | 26 / 23.3 / +0.56 |
| Online-only | 8 / 13.3 / -1.43 | 24 / 22.4 / +0.35 | 32 / 24.8 / +1.43 |

*χ*²(6) = 13.0, *p* = .043
