# Supplementary material for: Efficacy of a digital mental health program for university students: A randomized controlled trial comparing online and hybrid delivery
Source: Internet Interv. 2026 Sep 26;46:101002. doi: 10.1016/j.invent.2026.101002 (PMC13638797; doi:10.1016/j.invent.2026.101002)
Supplement: Supplementary file 3 [file mmc3.docx]

**Supplementary File S3.** Sensitivity analyses including program completion

**Model fit improvement with completion rate as a covariate**

Linear mixed-effects models including completion rate as a covariate demonstrated overall satisfactory fit across all outcomes (**Table S3.1**). Model adequacy was supported by consistent log-likelihood values, acceptable AIC and BIC indices, and substantial proportions of explained variance at both the fixed-effects and full-model levels. Overall, model fit indices indicate that the mixed-effects models adequately captured the longitudinal structure of the data across outcomes.

**Overall model fit and parsimony**

Across most outcomes, the inclusion of completion resulted in small but consistent improvements in model fit, as reflected by lower AIC and BIC values and slightly higher marginal R² values. These changes indicate a small improvement in model fit when completion was included. Importantly, conditional R² values and ICCs remained largely unchanged, suggesting that completion contributed primarily at the fixed-effects level.

**Mental health outcomes**

For total psychological distress (DASS-21), adding completion increased the variance explained by fixed effects from R² marginal = .061 to .067, alongside a modest improvement in AIC (ΔAIC ≈ −0.1). Similar patterns were observed for depression (ΔR² marginal = +.012) and, to a lesser extent, anxiety and stress. These results indicate a small incremental contribution of completion, particularly for depressive symptoms.

For global well-being (PERMAH total), the inclusion of completion led to a noticeable increase in marginal R² (from .018 to .027) and improved AIC, although the majority of variance remained attributable to between-person differences (ICC ≈ .77). Among PERMAH subscales, accomplishment showed the most pronounced improvement (ΔR² marginal ≈ +.014), suggesting greater sensitivity to engagement. Smaller but consistent gains were also observed for meaning, positive emotions, and relationships, whereas engagement and perceived health remained weakly explained by fixed effects overall. Loneliness and negative emotions showed negligible changes in model fit and explained variance, indicating that completion did not substantially contribute to explaining variability in these outcomes beyond time and group effects.

**Behavioural outcomes**

Sleep difficulties (ISI) showed a small increase in marginal R² (from .031 to .038) and a slight improvement in AIC, indicating that completion accounted for a limited but detectable proportion of variance beyond time and group effects. In contrast, alcohol use (AUDIT) exhibited minimal change in both fit indices and explained variance (ΔR² marginal ≈ +.001), while maintaining extremely high ICC values (> .85).

**Emotion regulation skills (ERSQ)**

For emotion regulation, the ERSQ total score showed a modest increase in marginal R² when completion was included (from .059 to .060), alongside a slight improvement in AIC. Although these changes were limited in magnitude, they were consistent with patterns observed across several ERSQ subscales.

Specifically, awareness, understanding, tolerance, clarity, and confrontation exhibited small increases in marginal R² (approximately +.001 to +.007), whereas bodily sensations and self-support showed minimal change. Notably, ICC values for confrontation and bodily sensations were lower than for other subscales, indicating greater within-person variability over time.

**Table S3.1**. Model fit indices for mixed-effects models including completion rate as covariate

| **Outcome** | **AIC** | **BIC** | **Log-Likelihood** | **R² Marginal** | **R² Conditional** | **ICC** |
| --- | --- | --- | --- | --- | --- | --- |
| **DASS-21 Total** | 14917.10 | 14983.08 | −7445.87 | 0.0667 | 0.6586 | 0.634 |
| Depression | 11420.52 | 11503.77 | −5706.21 | 0.0561 | 0.6372 | 0.616 |
| Anxiety | 10875.48 | 10961.45 | −5435.05 | 0.0372 | 0.6419 | 0.628 |
| Stress | 11049.72 | 11134.70 | −5521.68 | 0.0645 | 0.5666 | 0.537 |
| **ISI (Sleep)** | 12248.36 | 12327.51 | −6118.08 | 0.0377 | 0.6269 | 0.612 |
| **AUDIT** | 10162.74 | 10252.79 | −5080.73 | 0.0111 | 0.8577 | 0.856 |
| **PERMAH Total** | 17701.42 | 17750.51 | −8829.77 | 0.0267 | 0.7722 | 0.766 |
| Positive emotions | 11615.16 | 11695.21 | −5802.12 | 0.0198 | 0.7448 | 0.740 |
| Negative emotions | 11490.15 | 11570.72 | −5739.87 | 0.0348 | 0.6992 | 0.688 |
| Engagement | 11694.87 | 11774.27 | −5841.65 | 0.0129 | 0.6273 | 0.622 |
| Relationships | 12039.24 | 12117.07 | −6013.05 | 0.0132 | 0.7181 | 0.714 |
| Meaning | 12309.51 | 12386.05 | −6147.54 | 0.0192 | 0.7539 | 0.749 |
| Accomplishment | 11455.37 | 11536.14 | −5722.58 | 0.0392 | 0.7085 | 0.697 |
| Health | 11999.61 | 12077.66 | −5993.34 | 0.0106 | 0.7261 | 0.723 |
| Loneliness | 9407.03 | 9498.03 | −4703.53 | 0.0104 | 0.5737 | 0.569 |
| ERSQ Total | 16024.6924 | 16082.0115 | -7995.5185 | 0.0603 | 0.6411 | 0.618 |
| Awareness | 8554.4982 | 8649.7972 | -4279.4113 | 0.0406 | 0.5579 | 0.539 |
| Understanding | 8925.6756 | 9019.1045 | -4464.0650 | 0.0368 | 0.5729 | 0.557 |
| Modification | 8657.5398 | 8752.2882 | -4330.6568 | 0.0329 | 0.5345 | 0.519 |
| Tolerance | 9074.4675 | 9167.1089 | -4538.0672 | 0.0378 | 0.5521 | 0.534 |
| Acceptance | 8683.9461 | 8778.5186 | -4343.7720 | 0.0342 | 0.4997 | 0.482 |
| Clarity | 8926.7577 | 9020.2600 | -4464.6427 | 0.0353 | 0.6242 | 0.610 |
| Bodily sensations | 9231.7946 | 9323.5617 | -4616.2936 | 0.0279 | 0.4855 | 0.471 |
| Confrontation | 9100.0069 | 9192.3789 | -4550.7022 | 0.0457 | 0.4234 | 0.396 |
| Self-support | 8885.9576 | 8979.5252 | -4444.2753 | 0.0241 | 0.5169 | 0.505 |

**Effects of completion on outcomes and Time × Group interactions**

Linear mixed-effects models including the percentage of completion as a covariate were used to examine whether individual engagement contributed to outcome levels and to the interpretation of group-specific change trajectories over time (**Table S3.2**).

**Main effects of completion**

Completion showed significant main effects for a subset of outcomes, indicating that higher levels of program exposure were associated with more favorable scores independently of time and group assignment. Specifically, completion was significantly associated with depressive symptoms (F(1, 1219) = 7.98, p = .005), sleep difficulties (ISI) (F(1, 1249) = 4.58, p = .032), and overall well-being (PERMAH total) (F(1, 1080) = 6.21, p = .013). Within the PERMAH dimensions, significant effects of completion were observed for relationships (F(1, 1126) = 7.23, p = .007), meaning in life (F(1, 1093) = 4.61, p = .032), and accomplishment (F(1, 1130) = 12.12, p < .001), with a trend-level effect for perceived health (p = .088).

In contrast, completion was not significantly associated with total psychological distress, anxiety, stress, alcohol use (AUDIT), loneliness, or negative emotions, suggesting that these outcomes were less sensitive to variations in program exposure once time and group effects were accounted for.

Regarding emotion regulation, completion did not predict the ERSQ total score (F(1, 1202) = 0.30, p = .582). However, a significant main effect of completion was observed for the confrontation of situations subscale (F(1, 1390) = 6.50, p = .011), indicating that greater engagement was associated with increased tendency to face emotionally challenging situations. Other ERSQ subscales (awareness, modification, tolerance, acceptance, clarity, bodily sensations, and self-support) showed no significant main effects of completion.

**Time × Group interactions in the presence of completion**

Time × Group interactions remained significant across most outcomes after controlling for completion. Significant interactions were observed for total psychological distress and all DASS-21 subscales (all p < .001), sleep difficulties, overall well-being, several PERMAH dimensions (positive emotions and meaning), loneliness, negative emotions, the ERSQ total score, and most ERSQ subscales (awareness, modification, tolerance, acceptance, and clarity).

Notably, for several outcomes in which completion showed significant main effects (such as depression, ISI, and PERMAH total) the Time × Group interaction remained significant, suggesting that completion and group assignment contributed additively rather than redundantly to outcome changes. This indicates additive contributions of completion and group assignment to outcome changes.

Conversely, for certain emotion regulation dimensions, particularly confrontation, completion exerted a significant main effect whereas the Time × Group interaction was non-significant (p = .177). This suggests that improvements in this specific skill were more strongly related to individual engagement with the program than to experimental condition per se.

**Table S3.2.** Fixed effects of completion rate and completion × time interactions on outcome trajectories

| **Outcome** | **Completion F (df)** | **p** | **Time × Group F (df)** | **p** |
| --- | --- | --- | --- | --- |
| **DASS – Total** | 2.11 (1, 1203) | .146 | 11.05 (4, 1146) | < .001 |
| Depression | **7.98 (1, 1219)** | **.005** | 4.66 (4, 1152) | < .001 |
| Anxiety | 0.30 (1, 1210) | .582 | 7.13 (4, 1150) | < .001 |
| Stress | 0.07 (1, 1305) | .794 | 11.88 (4, 1190) | < .001 |
| **ISI** | **4.58 (1, 1249)** | **.032** | 5.74 (4, 1181) | < .001 |
| AUDIT | 0.59 (1, 1038) | .442 | 1.81 (4, 1100) | .125 |
| **PERMAH – Total** | **6.21 (1, 1080)** | **.013** | 3.40 (4, 1078) | .009 |
| Positive Emotions | 0.56 (1, 1096) | .453 | 4.40 (4, 1081) | .002 |
| Engagement | 0.90 (1, 1193) | .342 | 1.98 (4, 1117) | .096 |
| Relationships | **7.23 (1, 1126)** | **.007** | 0.56 (4, 1098) | .689 |
| Meaning | **4.61 (1, 1093)** | **.032** | 2.54 (4, 1082) | .038 |
| Accomplishment | **12.12 (1, 1130)** | **< .001** | 2.38 (4, 1093) | .050 |
| Health | 2.92 (1, 1123) | .088 | 2.18 (4, 1100) | .070 |
| **Loneliness** | 2.21 (1, 1250) | .137 | 3.39 (4, 1145) | .009 |
| **Negative Emotions** | 0.01 (1, 1147) | .926 | 7.84 (4, 1106) | < .001 |
| **ERSQ – Total** | 0.30 (1, 1202) | .582 | 7.38 (4, 1124) | < .001 |
| Awareness | 1.72 (1, 1270) | .190 | 6.41 (4, 1149) | < .001 |
| Modification | 0.00 (1, 1294) | .971 | 4.11 (4, 1162) | .003 |
| Tolerance | 2.79 (1, 1274) | .095 | 5.37 (4, 1150) | < .001 |
| Acceptance | 0.54 (1, 1335) | .463 | 4.34 (4, 1183) | .002 |
| Clarity | 0.24 (1, 1202) | .626 | 3.46 (4, 1120) | .008 |
| Sensations | 1.18 (1, 1346) | .277 | 1.98 (4, 1188) | .095 |
| **Confrontation** | **6.50 (1, 1390)** | **.011** | 1.58 (4, 1187) | .177 |
| Self-support | 0.11 (1, 1317) | .742 | 2.31 (4, 1178) | .056 |
