# Supplementary material for: Efficacy of a digital mental health program for university students: A randomized controlled trial comparing online and hybrid delivery
Source: Internet Interv. 2026 Sep 26;46:101002. doi: 10.1016/j.invent.2026.101002 (PMC13638797; doi:10.1016/j.invent.2026.101002)
Supplement: Supplementary file 4 [file mmc4.docx]

**Table S1.** Baseline equivalence across study groups on primary and secondary outcomes

| **Measure** | **F (2, 925)** | **p** | **η²p** | **ω²** | **Control** | **Hybrid** | **Online** |
| --- | --- | --- | --- | --- | --- | --- | --- |
| DASS-21 Total | 1.16 | .313 | 0.003 | 0.0003 | 22.84 (11.46) | 22.19 (11.71) | 22.63 (12.33) |
| DASS-21 Depression | 1.05 | .351 | 0.002 | 0.0001 | 8.23 (4.72) | 7.69 (4.65) | 8.24 (4.88) |
| DASS-21 Anxiety | 0.57 | .567 | 0.001 | 0.000 | 6.46 (4.08) | 6.10 (4.16) | 6.46 (4.44) |
| DASS-21 Stress | 1.11 | .332 | 0.002 | 0.0002 | 8.73 (3.88) | 8.40 (4.19) | 8.92 (4.25) |
| ISI | 0.94 | .392 | 0.002 | 0.000 | 11.22 (5.55) | 11.73 (5.68) | 11.79 (5.96) |
| **AUDIT** | **3.59** | **.028** | **0.008** | **0.006** | 4.10 (4.83) | 3.06 (4.00) | 3.48 (4.48) |
| PERMAH | 0.88 | .417 | 0.002 | 0.000 | 107.04 (28.02) | 108.76 (26.45) | 105.61 (28.87) |
| ERSQ | 0.72 | .487 | 0.002 | 0.000 | 61.47 (16.32) | 60.94 (15.96) | 60.00 (16.76) |

**Note.** Values are presented as means and standard deviations (SD). Statistically significant effects (p < .05) are shown in bold.
