# Supplementary material for: Efficacy of a digital mental health program for university students: A randomized controlled trial comparing online and hybrid delivery
Source: Internet Interv. 2026 Sep 26;46:101002. doi: 10.1016/j.invent.2026.101002 (PMC13638797; doi:10.1016/j.invent.2026.101002)
Supplement: Supplementary file 5 [file mmc5.docx]

**Supplementary Tables S2-S7. Post hoc analyses and estimated marginal mean differences**

**Table S2.** Estimated mean differences (Δ) for psychological distress (DASS-21)

| **Outcome** | **Time** | **Comparison** | **Δ (EMM)** | **95% CI** | **p (Bonf.)** |
| --- | --- | --- | --- | --- | --- |
| DASS total | T2 | Hybrid vs Control | −4.50 | [−6.67 ; −2.33] | **.002** |
|  | T2 | Online vs Control | −5.23 | [−7.08 ; −3.38] | **< .001** |
|  | T2 | Online vs Hybrid | −0.73 | [−2.89 ; 1.44] | 1.000 |
|  | T3 | Hybrid vs Control | −4.32 | [−6.68 ; −1.97] | **.012** |
|  | T3 | Online vs Control | −4.39 | [−6.47 ; −2.31] | **.001** |
|  | T3 | Online vs Hybrid | −0.07 | [−2.37 ; 2.23] | 1.000 |
| Depression | T2 | Hybrid vs Control | −1.39 | [−2.30 ; −0.48] | .099 |
|  | T2 | Online vs Control | −1.61 | [−2.38 ; −0.83] | **.002** |
|  | T2 | Online vs Hybrid | −0.21 | [−1.12 ; 0.70] | 1.000 |
|  | T3 | Hybrid vs Control | −1.30 | [−2.29 ; −0.31] | .361 |
|  | T3 | Online vs Control | −1.37 | [−2.24 ; −0.49] | .083 |
|  | T3 | Online vs Hybrid | −0.06 | [−1.03 ; 0.91] | 1.000 |
| Anxiety | T2 | Hybrid vs Control | −1.24 | [−2.04 ; −0.45] | .082 |
|  | T2 | Online vs Control | −1.60 | [−2.28 ; −0.93] | **< .001** |
|  | T2 | Online vs Hybrid | −0.36 | [−1.15 ; 0.43] | 1.000 |
|  | T3 | Hybrid vs Control | −1.22 | [−2.08 ; −0.36] | .214 |
|  | T3 | Online vs Control | −1.30 | [−2.07 ; −0.53] | **.032** |
|  | T3 | Online vs Hybrid | −0.08 | [−0.92 ; 0.76] | 1.000 |
| Stress | T2 | Hybrid vs Control | −1.86 | [−2.67 ; −1.05] | **< .001** |
|  | T2 | Online vs Control | −2.02 | [−2.71 ; −1.33] | **< .001** |
|  | T2 | Online vs Hybrid | −0.17 | [−0.98 ; 0.64] | 1.000 |
|  | T3 | Hybrid vs Control | −1.77 | [−2.66 ; −0.88] | **.003** |
|  | T3 | Online vs Control | −1.68 | [−2.47 ; −0.88] | **.001** |
|  | T3 | Online vs Hybrid | +0.10 | [−0.77 ; 0.97] | 1.000 |

**Table S3.** Estimated mean differences (Δ) for overall well-being (PERMAH total)

| **Time point** | **Comparison** | **Δ (EMM)** | **SE** | **95% CI** | **p (Bonf.)** |
| --- | --- | --- | --- | --- | --- |
| T2 | Control vs Hybrid | −3.80 | 2.74 | [−9.17 ; 1.56] | 1.000 |
| T2 | Control vs Online | −5.45 | 2.33 | [−10.02 ; −0.87] | .698 |
| T2 | Hybrid vs Online | −1.64 | 2.70 | [−6.93 ; 3.65] | 1.000 |
| T3 | Control vs Hybrid | −2.36 | 2.93 | [−8.10 ; 3.39] | 1.000 |
| T3 | Control vs Online | −2.86 | 2.55 | [−7.86 ; 2.14] | 1.000 |
| T3 | Hybrid vs Online | −0.50 | 2.86 | [−6.10 ; 5.09] | 1.000 |

**Table S4.** Estimated mean differences (Δ) for PERMAH subscales

| **Subscales** | **Time point** | **Comparison** | **Δ (EMM)** | **SE** | **95% CI** | **p (Bonf.)** |
| --- | --- | --- | --- | --- | --- | --- |
| Positive emotion | T2 | Control vs Hybrid | −0.66 | 0.57 | [−1.78 ; 0.45] | 1.000 |
|  | T2 | Control vs Online | −1.13 | 0.48 | [−2.08 ; −0.19] | .690 |
|  | T2 | Hybrid vs Online | −0.47 | 0.56 | [−1.57 ; 0.63] | 1.000 |
|  | T3 | Control vs Hybrid | −0.12 | 0.61 | [−1.31 ; 1.08] | 1.000 |
|  | T3 | Control vs Online | −0.52 | 0.53 | [−1.57 ; 0.52] | 1.000 |
|  | T3 | Hybrid vs Online | −0.41 | 0.60 | [−1.58 ; 0.77] | 1.000 |
| Engagement | T2 | Control vs Hybrid | −0.64 | 0.55 | [−1.72 ; 0.44] | 1.000 |
|  | T2 | Control vs Online | −1.24 | 0.47 | [−2.15 ; −0.32] | .301 |
|  | T2 | Hybrid vs Online | −0.59 | 0.55 | [−1.67 ; 0.48] | 1.000 |
|  | T3 | Control vs Hybrid | −0.14 | 0.60 | [−1.32 ; 1.03] | 1.000 |
|  | T3 | Control vs Online | −0.68 | 0.53 | [−1.73 ; 0.36] | 1.000 |
|  | T3 | Hybrid vs Online | −0.54 | 0.59 | [−1.70 ; 0.62] | 1.000 |
| Relationships | T2 | Control vs Hybrid | −0.77 | 0.63 | [−2.00 ; 0.46] | 1.000 |
|  | T2 | Control vs Online | −0.60 | 0.53 | [−1.64 ; 0.45] | 1.000 |
|  | T2 | Hybrid vs Online | 0.17 | 0.62 | [−1.04 ; 1.39] | 1.000 |
|  | T3 | Control vs Hybrid | −0.24 | 0.68 | [−1.56 ; 1.08] | 1.000 |
|  | T3 | Control vs Online | −0.03 | 0.59 | [−1.19 ; 1.13] | 1.000 |
|  | T3 | Hybrid vs Online | 0.21 | 0.66 | [−1.09 ; 1.51] | 1.000 |
| Meaning | T2 | Control vs Hybrid | −1.27 | 0.69 | [−2.61 ; 0.07] | 1.000 |
|  | T2 | Control vs Online | −1.21 | 0.58 | [−2.35 ; −0.07] | 1.000 |
|  | T2 | Hybrid vs Online | 0.06 | 0.68 | [−1.27 ; 1.38] | 1.000 |
|  | T3 | Control vs Hybrid | −0.77 | 0.73 | [−2.20 ; 0.67] | 1.000 |
|  | T3 | Control vs Online | −0.11 | 0.64 | [−1.36 ; 1.14] | 1.000 |
|  | T3 | Hybrid vs Online | 0.66 | 0.72 | [−0.75 ; 2.06] | 1.000 |
| Accomplishment | T2 | Control vs Hybrid | −0.64 | 0.54 | [−1.69 ; 0.41] | 1.000 |
|  | T2 | Control vs Online | −1.02 | 0.46 | [−1.91 ; −0.13] | 0.913 |
|  | T2 | Hybrid vs Online | −0.38 | 0.53 | [−1.42 ; 0.66] | 1.000 |
|  | T3 | Control vs Hybrid | −0.44 | 0.58 | [−1.58 ; 0.69] | 1.000 |
|  | T3 | Control vs Online | −0.47 | 0.51 | [−1.47 ; 0.53] | 1.000 |
|  | T3 | Hybrid vs Online | −0.03 | 0.57 | [−1.15 ; 1.09] | 1.000 |
| Health | T2 | Control vs Hybrid | 0.20 | 0.62 | [−1.02 ; 1.42] | 1.000 |
|  | T2 | Control vs Online | −0.34 | 0.53 | [−1.38 ; 0.69] | 1.000 |
|  | T2 | Hybrid vs Online | −0.54 | 0.62 | [−1.75 ; 0.67] | 1.000 |
|  | T3 | Control vs Hybrid | −0.66 | 0.67 | [−1.97 ; 0.65] | 1.000 |
|  | T3 | Control vs Online | −1.08 | 0.59 | [−2.23 ; 0.07] | 1.000 |
|  | T3 | Hybrid vs Online | −0.42 | 0.66 | [−1.71 ; 0.87] | 1.000 |
| Loneliness | T2 | Control vs Hybrid | 0.32 | 0.30 | [−0.28 ; 0.91] | 1.000 |
|  | T2 | Control vs Online | 0.26 | 0.26 | [−0.24 ; 0.76] | 1.000 |
|  | T2 | Hybrid vs Online | −0.06 | 0.30 | [−0.65 ; 0.53] | 1.000 |
|  | T3 | Control vs Hybrid | −0.34 | 0.33 | [−0.99 ; 0.32] | 1.000 |
|  | T3 | Control vs Online | 0.65 | 0.30 | [0.07 ; 1.23] | 1.000 |
|  | T3 | Hybrid vs Online | 0.98 | 0.33 | [0.34 ; 1.63] | 0.102 |
| Negative emotions | T2 | Control vs Hybrid | 1.65 | 0.54 | [0.60 ; 2.70] | 0.075 |
|  | T2 | Control vs Online | 2.11 | 0.46 | [1.21 ; 3.00] | **< .001** |
|  | T2 | Hybrid vs Online | 0.45 | 0.53 | [−0.59 ; 1.49] | 1.000 |
|  | T3 | Control vs Hybrid | 1.58 | 0.58 | [0.44 ; 2.72] | 0.239 |
|  | T3 | Control vs Online | 1.69 | 0.51 | [0.69 ; 2.70] | **0.033** |
|  | T3 | Hybrid vs Online | 0.12 | 0.57 | [−1.00 ; 1.23] | 1.000 |

**Supplementary Table S5.** Estimated mean differences (Δ) for behavioural outcomes

| **Scale** | **Time point** | **Comparison** | **Δ (EMM)** | **SE** | **95% CI** | **p (Bonf.)** |
| --- | --- | --- | --- | --- | --- | --- |
| ISI | T2 | Control vs Hybrid | 1.11 | 0.57 | [−0.00 ; 2.22] | 1.000 |
|  | T2 | Control vs Online | 1.46 | 0.48 | [0.51 ; 2.41] | 0.091 |
|  | T2 | Hybrid vs Online | 0.35 | 0.57 | [−0.76 ; 1.46] | 1.000 |
|  | T3 | Control vs Hybrid | 0.44 | 0.62 | [−0.77 ; 1.66] | 1.000 |
|  | T3 | Control vs Online | 1.27 | 0.55 | [0.20 ; 2.35] | 0.726 |
|  | T3 | Hybrid vs Online | 0.83 | 0.60 | [−0.35 ; 2.01] | 1.000 |
| AUDIT | T2 | Control vs Hybrid | 1.05 | 0.40 | [−0.25 ; 2.34] | .343 |
|  | T2 | Control vs Online | 0.88 | 0.34 | [−0.20 ; 1.97] | .352 |
|  | T2 | Hybrid vs Online | −0.16 | 0.39 | [−1.43 ; 1.10] | 1.000 |
|  | T3 | Control vs Hybrid | 0.52 | 0.42 | [−0.85 ; 1.89] | 1.000 |
|  | T3 | Control vs Online | 0.38 | 0.36 | [−0.79 ; 1.54] | 1.000 |

**Supplementary Table S6.** Estimated mean differences (Δ) for Emotion Regulation Skills (ERSQ total score)

| **Time point** | **Comparison** | **Δ (EMM)** | **SE** | **95% CI** | **p (Bonf.)** |
| --- | --- | --- | --- | --- | --- |
| T2 | Control vs Hybrid | −5.37 | 1.66 | [−8.62 ; −2.12] | **0.044** |
| T2 | Control vs Online | −4.90 | 1.41 | [−7.66 ; −2.14] | **0.019** |
| T2 | Hybrid vs Online | 0.47 | 1.65 | [−2.77 ; 3.70] | 1.000 |
| T3 | Control vs Hybrid | −0.86 | 1.82 | [−4.43 ; 2.71] | 1.000 |
| T3 | Control vs Online | −2.13 | 1.61 | [−5.28 ; 1.03] | 1.000 |
| T3 | Hybrid vs Online | −1.27 | 1.79 | [−4.77 ; 2.23] | 1.000 |

**Supplementary Table S7**. Estimated mean differences (Δ) for ERSQ subscales

| **Subscales** | **Time point** | **Comparison** | **Δ (EMM)** | **SE** | **95% CI** | **p (Bonf.)** |
| --- | --- | --- | --- | --- | --- | --- |
| Awareness | T2 | Control vs Hybrid | −0.60 | 0.24 | [−1.07 ; −0.12] | 0.483 |
|  | T2 | Control vs Online | −0.57 | 0.21 | [−0.97 ; −0.17] | 0.208 |
|  | T2 | Hybrid vs Online | 0.03 | 0.24 | [−0.44 ; 0.50] | 1.000 |
|  | T3 | Control vs Hybrid | −0.27 | 0.27 | [−0.80 ; 0.25] | 1.000 |
|  | T3 | Control vs Online | −0.44 | 0.24 | [−0.90 ; 0.02] | 1.000 |
|  | T3 | Hybrid vs Online | −0.17 | 0.26 | [−0.68 ; 0.35] | 1.000 |
| Modification | T2 | Control vs Hybrid | −0.51 | 0.25 | [−0.99 ; −0.03] | 1.000 |
|  | T2 | Control vs Online | −0.84 | 0.21 | [−1.26 ; −0.43] | **0.002** |
|  | T2 | Hybrid vs Online | −0.33 | 0.25 | [−0.82 ; 0.15] | 1.000 |
|  | T3 | Control vs Hybrid | −0.36 | 0.27 | [−0.89 ; 0.18] | 1.000 |
|  | T3 | Control vs Online | −0.59 | 0.24 | [−1.07 ; −0.12] | 0.528 |
|  | T3 | Hybrid vs Online | −0.24 | 0.27 | [−0.77 ; 0.29] | 1.000 |
| Understanding | T2 | Control vs Hybrid | −0.79 | 0.27 | [−1.31 ; −0.27] | 0.108 |
|  | T2 | Control vs Online | −0.41 | 0.23 | [−0.85 ; 0.04] | 1.000 |
|  | T2 | Hybrid vs Online | 0.38 | 0.27 | [−0.14 ; 0.90] | 1.000 |
|  | T3 | Control vs Hybrid | 0.28 | 0.29 | [−0.29 ; 0.86] | 1.000 |
|  | T3 | Control vs Online | 0.02 | 0.26 | [−0.50 ; 0.53] | 1.000 |
|  | T3 | Hybrid vs Online | −0.27 | 0.29 | [−0.84 ; 0.30] | 1.000 |
| Tolerance | T2 | Control vs Hybrid | −0.93 | 0.28 | [−1.47 ; −0.39] | **0.025** |
|  | T2 | Control vs Online | −0.64 | 0.23 | [−1.10 ; −0.18] | 0.229 |
|  | T2 | Hybrid vs Online | 0.29 | 0.28 | [−0.25 ; 0.83] | 1.000 |
|  | T3 | Control vs Hybrid | −0.14 | 0.31 | [−0.74 ; 0.45] | 1.000 |
|  | T3 | Control vs Online | −0.40 | 0.27 | [−0.93 ; 0.13] | 1.000 |
|  | T3 | Hybrid vs Online | −0.25 | 0.30 | [−0.84 ; 0.33] | 1.000 |
| Acceptance | T2 | Control vs Hybrid | −0.76 | 0.25 | [−1.24 ; −0.28] | **0.042** |
|  | T2 | Control vs Online | −0.57 | 0.21 | [−0.98 ; −0.15] | 0.252 |
|  | T2 | Hybrid vs Online | 0.19 | 0.25 | [−0.29 ; 0.68] | 1.000 |
|  | T3 | Control vs Hybrid | −0.31 | 0.28 | [−0.85 ; 0.23] | 1.000 |
|  | T3 | Control vs Online | −0.36 | 0.25 | [−0.84 ; 0.12] | 1.000 |
|  | T3 | Hybrid vs Online | −0.05 | 0.27 | [−0.58 ; 0.48] | 1.000 |
| Clarity | T2 | Control vs Hybrid | −0.47 | 0.27 | [−1.00 ; 0.05] | 1.000 |
|  | T2 | Control vs Online | −0.55 | 0.23 | [−0.99 ; −0.10] | 0.636 |
|  | T2 | Hybrid vs Online | −0.07 | 0.27 | [−0.60 ; 0.45] | 1.000 |
|  | T3 | Control vs Hybrid | 0.10 | 0.30 | [−0.48 ; 0.68] | 1.000 |
|  | T3 | Control vs Online | 0.00 | 0.26 | [−0.51 ; 0.51] | 1.000 |
|  | T3 | Hybrid vs Online | −0.10 | 0.29 | [−0.67 ; 0.47] | 1.000 |
| Sensations | T2 | Control vs Hybrid | −0.13 | 0.28 | [−0.68 ; 0.42] | 1.000 |
|  | T2 | Control vs Online | −0.21 | 0.24 | [−0.68 ; 0.26] | 1.000 |
|  | T2 | Hybrid vs Online | −0.08 | 0.28 | [−0.64 ; 0.47] | 1.000 |
|  | T3 | Control vs Hybrid | 0.01 | 0.32 | [−0.61 ; 0.63] | 1.000 |
|  | T3 | Control vs Online | −0.09 | 0.28 | [−0.64 ; 0.46] | 1.000 |
|  | T3 | Hybrid vs Online | −0.10 | 0.31 | [−0.70 ; 0.51] | 1.000 |
| Confrontation | T2 | Control vs Hybrid | −0.82 | 0.27 | [−1.35 ; −0.29] | **0.046** |
|  | T2 | Control vs Online | −0.59 | 0.23 | [−1.04 ; −0.14] | 0.385 |
|  | T2 | Hybrid vs Online | 0.23 | 0.27 | [−0.30 ; 0.76] | 1.000 |
|  | T3 | Control vs Hybrid | −0.21 | 0.30 | [−0.81 ; 0.40] | 1.000 |
|  | T3 | Control vs Online | −0.00 | 0.27 | [−0.54 ; 0.53] | 1.000 |
|  | T3 | Hybrid vs Online | 0.20 | 0.30 | [−0.39 ; 0.79] | 1.000 |
| Self support | T2 | Control vs Hybrid | −0.24 | 0.26 | [−0.75 ; 0.27] | 1.000 |
|  | T2 | Control vs Online | −0.63 | 0.22 | [−1.06 ; −0.20] | 0.164 |
|  | T2 | Hybrid vs Online | −0.39 | 0.26 | [−0.90 ; 0.12] | 1.000 |
|  | T3 | Control vs Hybrid | 0.09 | 0.29 | [−0.48 ; 0.66] | 1.000 |
|  | T3 | Control vs Online | −0.24 | 0.26 | [−0.75 ; 0.26] | 1.000 |
|  | T3 | Hybrid vs Online | −0.34 | 0.29 | [−0.90 ; 0.22] | 1.000 |
